# Antibody targeting tumor-derived soluble NKG2D ligand sMIC provides dual co-stimulation of CD8 T cells and enables sMIC^+^ tumors respond to PD1/PD-L1 blockade therapy

**DOI:** 10.1186/s40425-019-0693-y

**Published:** 2019-08-26

**Authors:** Jinyu Zhang, Pablo Saenz-lopez Larrocha, Bin Zhang, Derek Wainwright, Payal Dhar, Jennifer D. Wu

**Affiliations:** 1 Department of Microbiology and Immunology, Medical University of South Carolina, Charleston, SC USA; 2 Feinberg School of Medicine, Northwestern University, Chicago, IL 60611 USA

**Keywords:** NKG2D, Soluble MHC I chain related molecule (sMIC), Cancer, PD1 blockade, Immunotherapy

## Abstract

**Background:**

Insufficient co-stimulation accounts for a great deal of the suboptimal activation of cytotoxic CD8 T cells (CTLs) and presumably unsatisfactory clinical expectation of PD1/PD-L1 therapy. Tumor-derived soluble NKG2D ligands are associated with poor clinical response to PD1/PD-L1 blockade therapy in cancer patients. One of the mostly occurring tumor-derived soluble NKG2D ligands, the soluble MHC I chain related molecule (sMIC) can impair co-stimulation to CD8 T cells. We investigated whether co-targeting sMIC can provide optimal co-stimulation to CTLs and enhance the therapeutic effect of PD1/PD-L1 blockades.

**Methods:**

Single agent therapy of a PD1/PD-L1 blockade antibody or a sMIC-targeting non-blocking antibody or a combination therapy of the two antibodies were implied to well-characterized pre-clinical MIC/sMIC^+^ tumor models that closely resemble the NKG2D-mediated oncoimmune dynamics of MIC^+^ cancer patients. Therapeutic efficacy and associated effector mechanisms were evaluated.

**Results:**

We show that antibody co-targeting sMIC enables or enhances the response of sMIC^+^ tumors to PD1/PD-L1 blockade therapy. The therapy response of the combination therapy was associated with enhanced antigen-specific CD8 T cell enrichment and function in tumors. We show that co-targeting sMIC with a nonblocking antibody provides antigen-specific CD8 T cells with NKG2D and CD28 dual co-stimulation, in addition to elimination of inhibitory signals, and thus amplifies antigen-specific CD8 T cell anti-tumor responses.

**Conclusion:**

Our findings provide the proof-of-concept rationale and previously undiscovered mechanisms for co-targeting sMIC to enable and enhance the response to PD1/PD-L1 blockade therapy in sMIC^+^ cancer patients.

**Electronic supplementary material:**

The online version of this article (10.1186/s40425-019-0693-y) contains supplementary material, which is available to authorized users.

## Introduction

The generation of potent cytotoxic CD8 T cells (CTLs) that are capable of destroying tumor cells requires optimal TCR stimulation along with the provision of co-stimulatory signals, in addition to minimize co-inhibitory signaling, such as PD-1 immune checkpoint blockades [1–3]. Insufficient co-stimulation accounts for a great deal of the suboptimal activation and maintenance of tumor-killing CD8 T cells [2, 3]. Many strategies have been employed to manipulate the co-stimulatory signals to induce maximum T cell anti-tumor capacity. For instance, a great deal of efforts has been made in manipulating the canonical co-stimulatory molecule CD28 and the activation induced TNF-R superfamily costimulatory molecules [4] . However, each of these co-stimulatory pathways faces different challenges as their expression is often influenced by the “second wave” of T cell activation [1, 4, 5]. More critically, due to unrestricted expression of these molecules on activated lymphocytes (e.g. CD4 T, B cells) other than CTLs, precipitation of excessive systemic inflammation and invigorating silent autoimmunity are the inherent dangers of these immunomodulation strategies [4].

NKG2D, an activating receptor expressed by all human NK cells, is a constitutively expressed co-stimulatory receptor for all human CD8T, NKT cells and subsets of γδT cells [6–11]. Similar to the canonical co-stimulatory molecule CD28 and activation-induced TNF-R superfamily of costimulatory molecules, NKG2D co-stimulation synergizes with CD3/TCR signaling independent of CD28 [9, 11]. Different from these well-studied co-stimulatory molecules, expression of NKG2D expression is constitutive, independent of T cell activation or functional status in human CD8 T cells. More importantly, NKG2D is not found on CD4 T cells or B cells under normal physiological condition [6–12]. In humans, NKG2D is activated through binding to the family of ligands of the MHC I Chain related molecules A and B (MICA and MICB, collectively termed MIC) and the HCMV UL-16 binding proteins (ULBPs) [8]. These ligands are often only induced to express on the surface of cells that are under oncogenic or environmental insults, but not present on the healthy cells. Therefore, NKG2D is considered as the immune surveillance receptor to eliminate abnormal cells [8].

Among the family of human NKG2D ligands, MICA and MICB are the most frequently and broadly expressed ligands on human solid tumors [13]. The two molecules MICA and MICB share similar, if not identical, immune stimulatory function but present variation in expression in tumors, presumably due to the evolutionary process [14, 15]. Malignant human tumors often evade the NKG2D immunity by releasing the soluble form of MIC (sMIC), through the process of proteolytic shedding [16–19]. sMIC are highly immune suppressive by multiple mechanisms, such as disturbing NK cell homeostatic maintenance and function [16, 20, 21], facilitating expansion of myeloid derived suppressor cells (MDSC) in tumor microenvironment [22], and more profoundly, impairing antigen-specific CD8 T cell activation via down-regulating NKG2D co-stimulation and destabilizing the TCR/CD3 signaling molecule CD3ζ through activation of caspase 8 pathway [20, 23]. Elevated levels of serum sMICA or sMICB are associated with tumor progression and metastasis [24, 25].

High levels of circulating soluble NKG2D ligands are associated with poor clinical outcome of PD1/PD-L1 blockade therapy [26]. Given this clinical observation and immune suppressive effect of sMIC, in the current study, we tested the hypothesis that antibody targeting sMIC could enhance the therapeutic efficacy of PD-1/PD-L1 blockade. With well characterized preclinical models, we show that targeting sMIC with a nonblocking monoclonal antibody enables sMIC^+^ tumors response to PD1/PD-L1 therapy. We demonstrate that co-targeting sMIC with a PD1/PD-L1 blockade profoundly enhances tumor-infiltrated CD8 T cells intrinsic function in general, enhances the function and proliferative ability of antigen-specific CD8 T cells in tumors. Intriguingly, we found that targeting sMIC provides enhanced and sustained NKG2D and CD28-mediated dual co-stimulation and amplifies TCR activation in CD8 T cells. Our findings provide the proof-of-concept rationale and previously undiscovered mechanisms for translating a novel combination immunotherapy to enhance the response to PD1/PD-L1 blockade therapy in sMIC^+^ cancer patients.

## Material and methods

### Animals and antibody therapy

All experimental procedures were approved by the Institutional Animal Care and Use Committee (IACUC) protocol at Medical University of South Carolina and Northwestern University. All mice are maintained at respective Institutional animal facility under specific pathogen-free conditions. Generation and characterization of the TRAMP/MICB mice have been previously described [27]. Briefly, the human MICB was overexpressed in the prostate under the hormone-sensitive rat probasin (rPB) promoter to generate MICB/B6 mice. The MICB/B6 mice were bred with TRAMP mice to generate TRAMP/MICB mice which have been shown to recapitulate the NKG2D-mediated onco-immune dynamics of MIC^+^ cancer patients [27]. TRAMP/MICB male mice of 26 to 28 week-old were assigned into four cohorts with similar distribution of serum sMIC (Additional file 1: Figure S1) receiving therapy with intraperitoneal (i.p.) injection of sMIC-targeting nonblocking monoclonal antibody B10G5 [28] or anti-PD-L1 (clone 10F.9G2, BioXCell) antibody or isotype control IgG (cIgG) respectively at a respective dose of 4.0 mg/kg body weight twice weekly. For NK depletion, anti-NK1.1 (PK136) mAb (100 μg/mouse) were given 1 day before therapy and thereafter twice weekly as well together with therapeutic antibodies. All animals were treated for 8 weeks before euthanization which was designed for the study end point. Each study was repeated for three times unless otherwise specified.

### Antibody reagents and flow cytometry

Single cell suspension from spleen, draining lymph nodes (dLN), non-dLN or tumor were prepared as previous description [28]. Combination of the following antibody was used for cell surface or intracellular staining to define populations of NK, CD8, and subsets of CD4 T cells: CD3e (clone 145-2c11), CD8a (clone 53–6.7), CD4 (clone GK1.5), NK1.1 (clone PK136), NKG2D (clone CX5), CD44 (clone eBio4B10), CD11c (clone N418), MHCII (clone M5/114.15.2), CD80 (clone 16-10A1), CD86 (clone PO3) and CD40 (clone 1C10). For ex vivo re-stimulation, single cell suspension of freshly isolated splenocytes, LNs or TILs were cultured in complete RPMI 1640 medium containing 50 ng/mL PMA and 500 ng/mL Ionomycin for 6 h and analyzed by intracellular staining with antibodies specific to IFNγ (XMG1.2). All antibodies and the corresponding isotype controls were fluorochrome conjugated and were purchased from Biolegend, eBioscience or BD Biosciences. Multicolored Flow cytometry analyses were performed on an LSR II (BD). Data were analyzed with FlowJo X software (Tree Star).

### Ex vivo antigen-specific T cell stimulation

Single suspended splenocytes from TCR-I transgenic mice were injected *i.v.* into animals (1x10^6^cells/mouse) that were received B10G5, anti-PD-L1 antibody, antibody cocktail, or control IgG therapy at 4.0 mg/kg body weight for each mouse. Animals were sacrificed at indicated time points to assess TCR-I T cell in vivo frequency with TCR-I-specific H-2D^b^/TAg epitope I-tetramer (D^b^/I-tetramer) [29]. To assay antigen-specific CD8^+^ T cell response, single cell suspension of splenocytes, tumor-draining lymph nodes (dLN) and tumor infiltrated lymphocytes (TILs) were stimulated overnight with 0.5 μM TAg epitope I peptide (SAINNYAQKL) and assayed by intracellular IFNγ staining of CD8^+^ or D^b^/I-tetramer^+^ T cells.

### In vivo proliferation assay

For in vivo proliferation assays, splenocytes from TCR-I transgenic mice were suspended at 1 × 10^7^/ml in PBS/0.1% BSA and labeled with 5 μM CFSE (Biolegend, San Diego, CA, USA) for 10 min at 37 °C. Cells were then washed for three times in PBS, finally resuspended in PBS, and injected by i.v at a dose of 5 × 10^6^ cells per mouse. After 14 days, isolation of spleens, dLNs and TILs from recipient mice were harvested, and the intensity of CFSE staining was measured among CD8^+^ D^b^/I-Tetramer^+^ T cells by flow cytometry.

### Tissue collection

Mouse blood was collected via tail bleeding before therapy or via cardiac puncture after euthanization. Serum was separated from blood by centrifugation. Splenocytes, draining lymph nodes (dLN), non-draining lymph nodes and partial of prostate tumors were directly meshed for isolation of TILs were collected for immunological analyses. Partial of prostate, lung, liver, kidney, pancreas, and colons were collected and fixed in 10% neutral fixation buffer followed by paraffin embedment for pathological and histological analyses.

### Serum sMIC detection

Serum levels of sMICB from experimental mice were assessed using Duoset MICB Sandwich ELISA kit (Cat. DY1599) from R&D Systems according to manufacturer’s instruction. Serum was diluted 1:20 in PBS. Each assay was run in triplicates.

### TCR-specific human T cells stimulation assay

Human CD8 T cells were seeded in anti-CD3 (1 μg/ml, BD Biosciences) pre-coated 96-well plates and cultured with conditions where indicated with the following reagents: 1) 1 μg/ml soluble anti-CD28 antibody (Biolegend); 2) 100 ng/ml of soluble recombinant MICB (Sino Biologicas); 3) 100 ng/ml of B10G5. IFNγ production was assayed by intracellular staining after 24 h of culture (BD IFNγ staining Kits).

For assessing antigen-specific CD8 T cell response, human tyrosinase-specific HLA-A_2_-restricted TIL13831 was co-cultured o/n with the HLA-A2^+^ T2-A2 cells (Generous gifts of Dr. Rubinstein at the Medical university of South Carolina) under indicated condition before functional assay. The tyrosinase peptide_369–377_ was purchased from AnaSpec (Fremont, CA). After overnight culture, activation of TIL13831 was assessed by intracellular staining for IFNγ, TNFα, and CD107a (degranulation).

### Statistical analysis

All results are expressed as the mean ± SEM. Mouse and sample group were *n* > 5, unless otherwise indicated. Data were analyzed using unpaired t-test, and treatment differences were considered significant at *P* values< 0.05. Kaplan-Meier survival curves were generated using GraphPad Prism software.

## Results

### Antibody targeting sMIC/MIC enables and enhances tumors respond to anti-PD1/PD-L1 therapy

Tumor-derived sMIC suppresses anti-tumor immunity through impairing NK and CD8 T cell function and facilitating the expansion of MDSCs in tumor microenviroment [17, 20, 22, 27]. High levels of serum sMIC either at baseline or during therapy correlate with poor response to PD1/PD-L1 blockade therapy [26, 30]. We thus sought to investigate whether targeting sMIC can enhance tumor response to PD1/PD-L1 blockade therapy in pre-clinical models. With the knowledge that rodents do not express orthologs of human MIC and that human MICB serves as a functional ligand for mouse NKG2D, we generated a bi-transgenic TRAMP/MICB mouse that recapitulates the onco-immunological characteristics of human MIC^+^ cancer patients in that: i) MIC is specifically expressed in a given organ and concurrently expressed with oncogenic insults; ii) tumor releases sMIC during disease progression; iii) elevated levels of circulating sMIC correlate with more immune suppressive phenotype and more aggressive diseases [27].

**Fig. 1:**
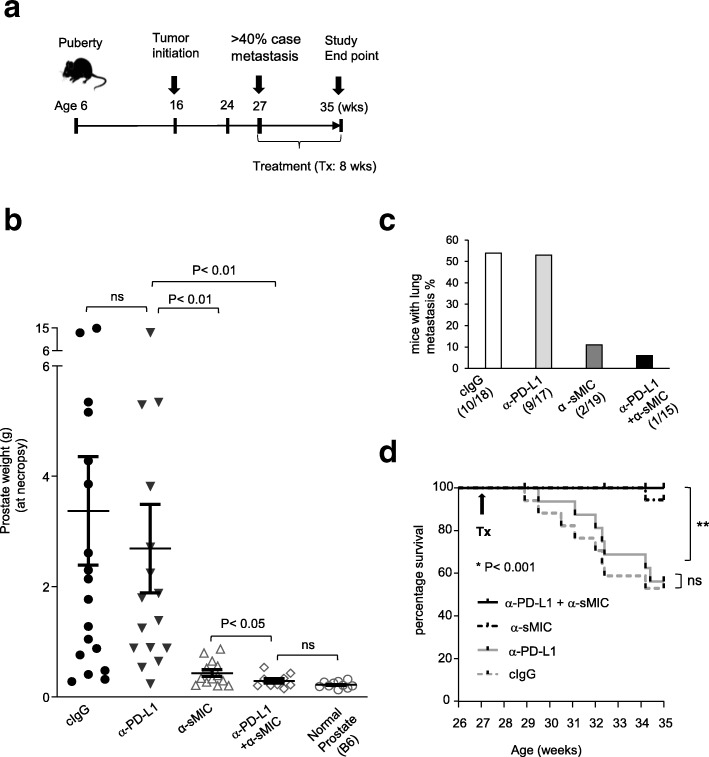
Therapeutic efficacy of antibody cocktail composed of the sMIC-targeting antibody B10G5 and an anti-PD-L1 antibody in the autochthonous bi-transgenic TRAMP/MICB mice. **a**, Depiction of the therapy schema. Cohorts of 27 to 29-week-old TRAMP/MICB mice (male) were assigned to four therapy groups according to similar distribution of serum levels of sMIC for the four defined therapy. All therapies were given twice weekly for 8 weeks. **b**, Prostate organ weight, which reflects tumor weight in situ, of the animals at necropsy after receiving an 8-week duration of specific therapy. **c**, Incidence of lung metastasis in animals of each therapeutic group after a duration of 8-week therapy. Due to multiple focal micrometastasis in distant organs, quantitation of micrometastasis in each organ is not achievable. **d**, Kaplan-Meier survival at the designated study point (end of 8-week therapy). ns, not statistically significant

To investigate whether targeting sMIC enables or enhances the response of MIC^+^ tumors to PD1/PD-L1 blockade therapy in TRAMP/MICB mice, we evaluated serum levels of sMIC in animals of ages between 27 to 29-weeks old when tumors are easily palpable in the abdominal. In accordance with our previous work, more than 50% of TRAMP/MICB mice would have invasive tumors with distant metastases in draining lymph nodes or lungs at 27 weeks of age [27]. These animals represented a subject population with heterogeneous diseases and varied levels of circulation serum sMIC [27, 31]. Given that higher levels of serum sMIC generally reflect more advanced diseases in TRAMP/MICB mice [27], we assigned these animals into four therapeutic groups, with the consideration that each group consists of animals with similar distribution of serum sMIC (Additional file 1: Figure S1). The four therapies include: an anti-PD-L1 antibody, a well-described sMIC-targeting monoclonal antibody (mAb) B10G5, an antibody cocktail composed of anti-PD-L1 mAb and B10G5, and control IgG (Fig. 1a). Consistent with our previous findings [28], all animals responded to the sMIC-targeting antibody B10G5, as demonstrated by the significantly decreased tumor weight (reflected as prostate organ weight) and reduced incidence of distant metastasis as compared to control IgG-treated animals (Fig. 1b and c). Animals that received anti-PD-L1 antibody therapy generally did not elicit a signficant beneficial response (Fig. 1b and c). Strikingly, animals subjected to combination therapy of the anti-PD-L1 mAb and B10G5 showed a significant decrease in tumor weight as compared to all other groups (Fig. 1b). Combination treatment also significantly decreased the incidence of lung metastasis and improved overall survival (Fig. 1c and d). These data demonstrate that antibody targeting sMIC resulted in the response of tumors, otherwise unresponsive, to PD1/PD-L1 blockade therapy.

As the mAb B10G5 recognizes both sMIC and membrane-bound MIC as we have described previously [28], we thus sought to confirm that sMIC indeed negatively impacts tumor response to PD1/PD-L1 therapy, with a sMIC-expressing syngeneic tumor model (Additional file 1: Figure S2). Of note, tumors express strictly membrane-bound MIC rarely arose in animals [27, 32], which makes it unfeasible to address how much impact membrane-bound MIC has on PD1/PD-L1 blockade therapy. We compared tumor response to anti-PD-L1 antibody in the well-described syngeneic TRAMP-C2 and sMICB-overexpressing TRAMP-C2-sMICB mouse prostate tumor models. In agreement with our findings in TRAMP/MICB mice, animals bearing TRAMP-C2-sMICB tumors elicited impaired response to anti-PD-L1 antibody therapy as compared to mice bearing TRAMP-C2 tumors (Additional file 1: Figure S2b-d).

To substantiate the observation that targeting sMIC enables/enhances the response of sMIC^+^ tumors to PD1/PD-L1 blockade treatment, we randomize mice bearing TRAMP-C2-sMICB tumors into four treatment groups of control, monotherapy with B10G5 or a PD1 blocking antibody, and the combination therapy (Additional file 1: Figure S3). Consistent with the outcome in TRAMP/MICB mice, combination therapy resulted in better outcome than monotherapy of B10G5 or the PD1 blockade antibody (Additional file 1: Figure S3).

### Concurrent therapy of antibody targeting sMIC and anti-PD-L1 mAb cooperatively augments CD8 T cells the intrinsic ability to be activated

**Fig. 2:**
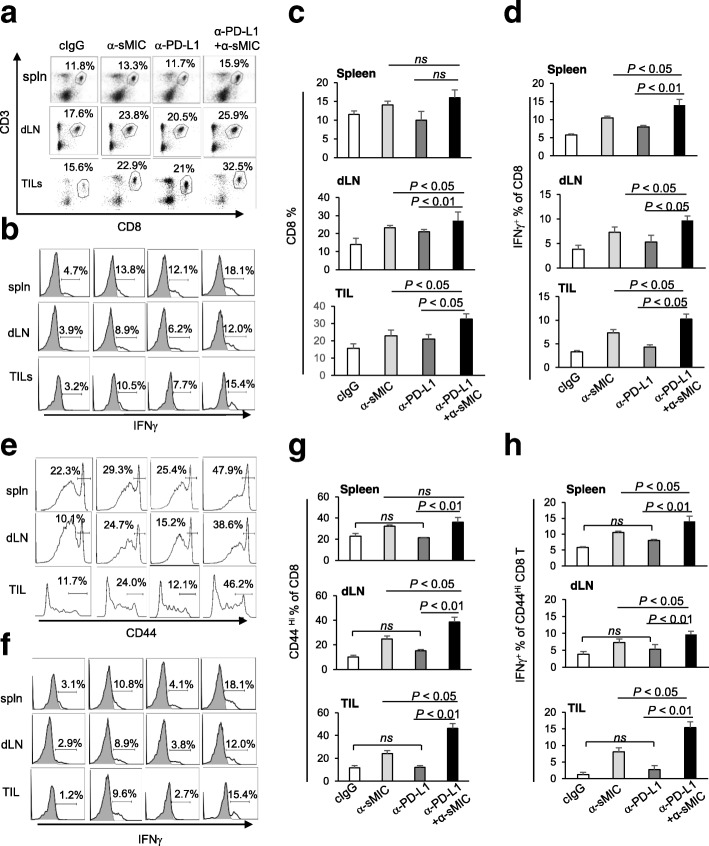
Combination therapy of sMIC-targeting mAb B10G5 and anti-PD-L1 antibody cooperatively enhances anti-tumor potential of CD8^+^ T cells. **a** and **c**, Representative dot plots and summary data from all animals present that cocktail therapy significantly enriched CD8 T cell in draining LN (dLN) and tumor infiltrates. **b** and **d**, Representative histograms (**c**) and summary data (**d**) of CD8 T cell IFNγ production in response to SV40TAg re-stimulation. **e** and **g**, Representative histograms (**e**) and summary data (**f**) of CD44^Hi^ CD8 T cell population. **f** and **h**. Representative histograms (**g**) and summary data (**h**) of IFNγ production by CD44^Hi^ CD8 T cells in response to PMA/ionomycin stimulation. Spln, spleen. dLN, tumor-draining lymph node, TIL, tumor infiltrated lymphocytes. ns, not significant

Combination therapy of anti-PD-L1 mAb and B10G5 targeting sMIC significantly augmented CD8 T cell mediated anti-tumor immunity. Although combination therapy did not significantly impact the population of CD8 T cells in peripheral lymphoid tissues, such as spleen, CD8 T cell population in the tumor-draining lymph nodes (dLN) and tumor was significantly increased (Fig. 2a, c). Consistent with our previous findings [28], B10G5 also augmented antigen-specific CD8 T cell effector functions in peripheral lymphoid tissue and tumor site (Fig. 2b, d). Monotherapy with anti-PD-L1 mAb did not significantly enhance antigen-specific CD8 T cell function as measured by ex vivo response to stimulation by TRAMP-specific SV40TAg peptide. However, combination therapy of the anti-PD-L1 mAb and B10G5 remarkably enhanced the antigen-specific responsiveness of CD8 T cells as compared to B10G5 single agent therapy (Fig. 2b, d). The augmented therapy effect of combined reagents was further demonstrated by a significant increase in the CD44^hi^ CD8 T cell compartments in the spleen, dLN, and tumors and the significant increase in their cell intrinsic ability to produce IFNγ (Fig. 2e-h). These results suggest a potential synergistic effect of B10G5 targeting sMIC and the anti-PD-L1 mAb therapy.

### Therapy of anti-PD-L1 mAb and targeting sMIC resulted in enhanced and sustained intra-tumoral antigen specific CD8 T cell anti-tumor ability

**Fig. 3:**
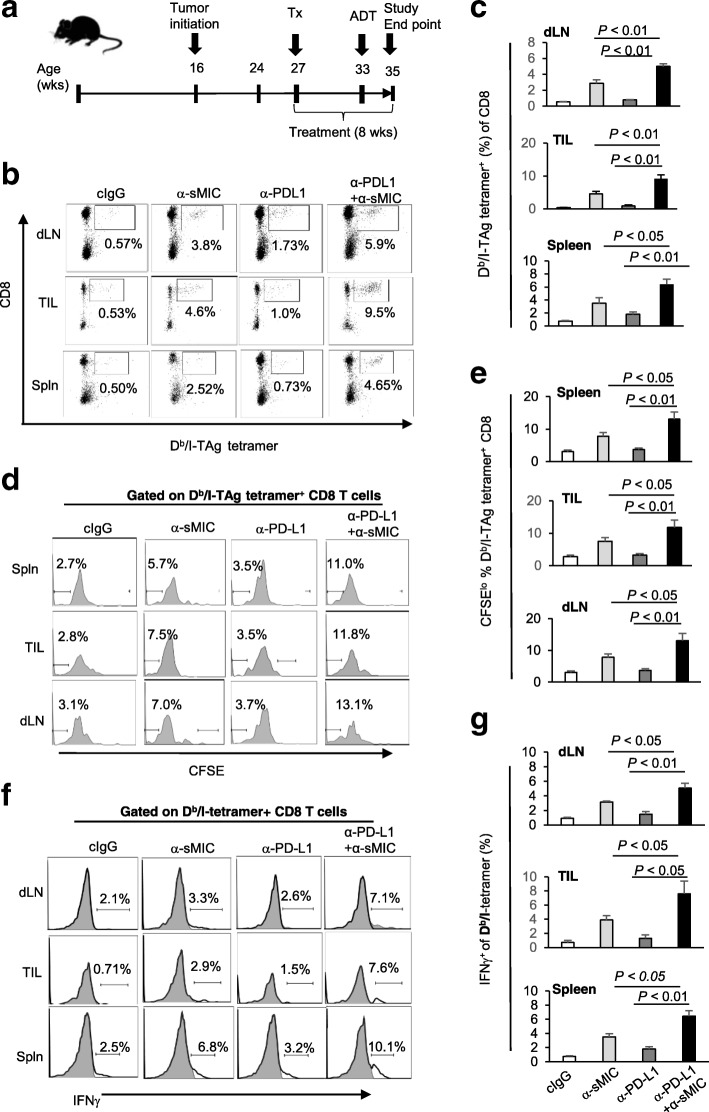
Anti-PD-L1 antibody in combination with sMIC-targeting mAb B10G5 cooperatively enhances antigen-specific CD8 T cell anti-tumor responses. **a**, Depiction of experiment schema. CFSE-labeled tumor antigen SV40TAg-specific TCR-I CD8 T cells were transferred into TRAMP/MICB mice that have received 4-week duration of therapy, which was continued post transfer of TCR-I CD8 T cells. Data shown are 14-day post-transfer of CFSE-labeled TCR-I CD8 T cells. **b** and **c**, Representative dot plots (**b**) and summary data (**c**) demonstrating the percentage of D^b^/I-tetramer^+^ SV40TAg-specific CD8 T cells in dLN, tumor infiltrates, and spleen. **d** and **e**, Representative histograms (**d**) and summary data (**e**) demonstrating proliferation of SV40Tag-specific CD8 T cells represented by CFSE^lo^ population. **f** and **g**, Representative histograms (**f**) and summary data (**g**) demonstrating the response of D^b^/I-tetramer^+^ SV40TAg-specific CD8 T cells to ex vivo SV40TAg peptide re-stimulation as measured by IFNγ production

To investigate whether anti-PD-L1 mAb and B10G5 combination therapy enhances CD8 T cell anti-tumor response in an antigen-specific manner, we utilized the availability of SV40TAg-specific T cell receptor (TCR) transgenic (TCR-I) mice [29]. Of note, tumors in TRAMP/MICB mice were driven by SV40TAg through interrupting p53 and Rb signaling [33]. CD8 T cells from the TCR-I mice (thereafter TCR-I CD8 T cell) bear SV40TAg-specific TCR and can be detected by SV40TAg peptide I-specific D^b^/I-tetramer. We adoptively transferred CFSE-labeled TCR-I CD8 T cell into mice that have received four-week duration of treatment as depicted in Fig. 3a and analyzed the sustainability of these TCR-I CD8 T cells on day 14 post-adoptive transfer. Normally, adoptively transferred antigen-specific TCR-I CD8 T cells fail to be sustained after their initial expansion in TRAMP or TRAMP/MICB mice due to clonal deletion [28, 34]. While monotherapy with anti-PD-L1 mAb presented a marginal effect on sustaining D^b^/I-tetramer^+^ TCR-I CD8 T cells in the dLN, tumor, or spleen, B10G5 therapy consistently sustained the adoptively transferred TCR-I CD8 T cells with high frequency in the tumor as we have previously shown [28] (Fig. 3b, c). Remarkably, combination therapy of anti-PD-L1 and B10G5 further significantly enhanced the sustainability of TCR-I CD8 T cells in comparison to B10G5 monotherapy (Fig. 3b, c). The subsequent CFSE dilution assay confirmed that only therapy with mAb B10G5 or combination therapy provoked an expansion of SV40TAg-specific TCR-I CD8 T cells represented by an elevation in the percentage of CFSE^lo^ D^b^/I-tetramer^+^ CD8 T cells. However, combination therapy remarkably enhanced the expansion as compared to B10G5 monotherapy (Fig. 3d, e). Expansion of antigen-specific CD8 T cells in the tumor and draining lymph nodes is considered the hallmark for activation of antigen-specific CD8 T cells. In support of this concept, combination therapy resulted in significantly enhanced IFNγ production by D^b^/I-tetramer^+^ CD8 T cells in response to TAg-peptide re-stimulation (Fig. 3f, g). Together, these data demonstrate an important effector mechanism for cooperative therapeutic effect of anti-PD-L1 mAb and B10G5.

### Combination therapy of anti-PD-L1 mAb and B10G5 markedly enhances the co-stimulatory potential of dendritic cells (DCs)

**Fig. 4:**
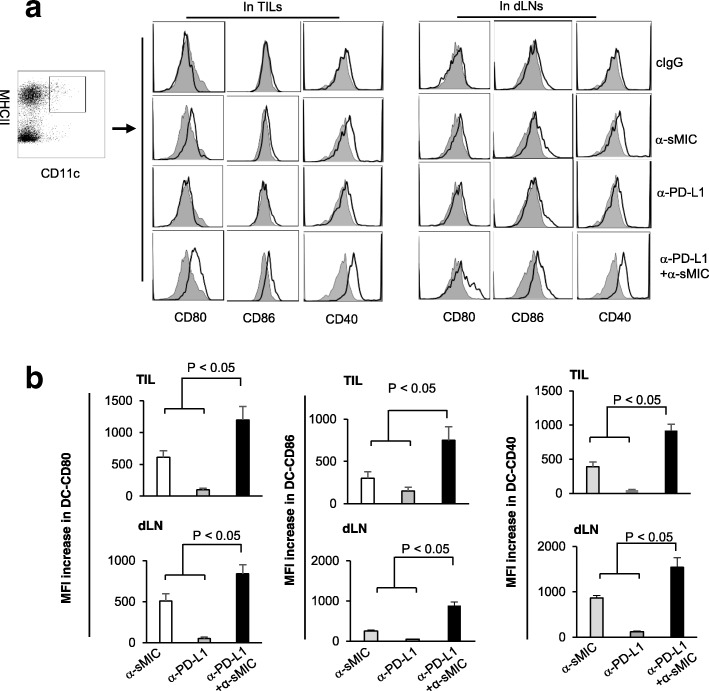
Combination therapy of anti-PD-L1 antibody and sMIC-targeting mAb B10G5 cooperatively increase DC activation (CD40) and the expression of co-stimulatory molecules CD80 and CD86 in tumor sites. **a**, Representative histograms from flow cytometry analyses of CD40, CD80 and CD86 expression on DCs from tumor-draining lymph nodes and tumor beds. Gray filled profiles, control isotype staining. Open dark profiles, antibody to specific DC surface molecules. **b**, Summary data of the increase in mean fluorescence intensity (MFI) of CD80, CD86, and CD40 on DCs

We have previously shown that single agent B10G5 enhances DC activation in tumor draining lymph nodes and increases the expression of DC co-stimulatory molecule CD80 and CD86 [28]. Combination therapy of anti-PD-L1 mAb and B10G5 further significantly increased DC surface expression of the co-stimulatory molecule CD80 and CD86 as well as the DC activation molecule CD40 (Fig. 4a and b). Given that CD80 and CD86 engagement with CD28 on CD8 T cells amplifies TCR/CD3 signaling, these data indicate that inhibition of the PD1/PD-L1 pathway with co-targeting sMIC could potentially facilitate a more vigorous CD28-mediated co-stimulatory signal delivered to antigen-specific CD8 T cells for the sustained anti-tumor activity.

### Targeting sMIC stabilizes NKG2D and upregulates CD28 expression on tumor-infiltrating CD8 T cells

**Fig. 5:**
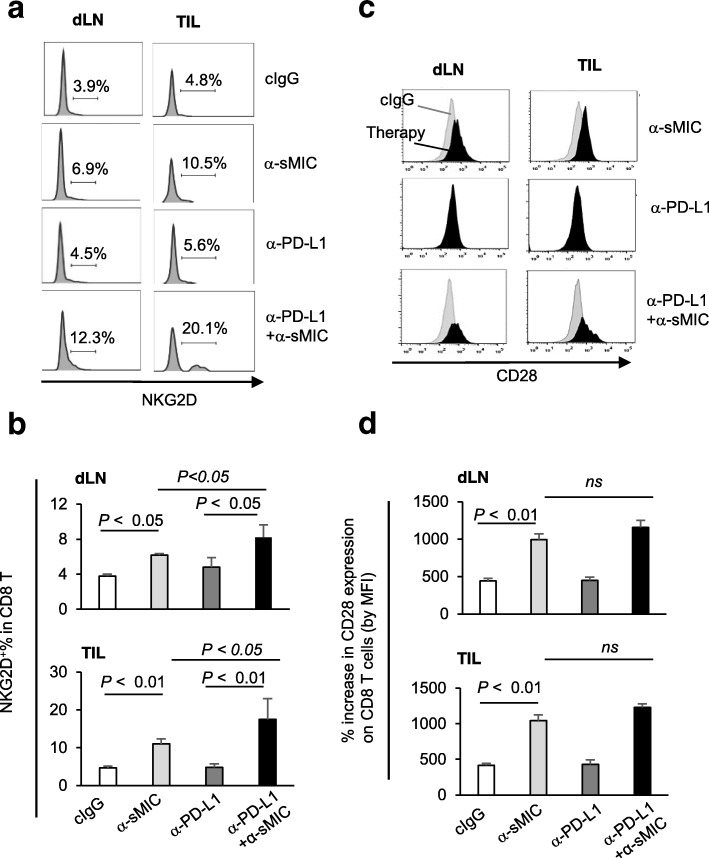
Targeting sMIC increases co-stimulatory molecules on CD8 T cells in tumor draining LN and tumor infiltrates. **a** and **b**, Representative histograms (**a**) and summary data (**b**) demonstrating NKG2D expression on CD8 T cells. **c** and **d**, Representative histogram overlay (**c**) and summary data of mean fluorescence intensity (MFI) (**d**) demonstrating CD28 expression on CD8 T cells. Grey profiles in (**c**) are CD28 expression in CD8 T cells from cIgG treated animals. Black profiles in (**c**) are CD28 expression in CD8 T cells from animals receiving respective therapy

NKG2D is a co-stimulatory molecule on CD8 T cells that functions non-redundantly of CD28 [12, 35, 36]. Tumor-derived sMIC was shown to downregulate NKG2D expression in cancer patients and subvert NKG2D-co-stimulation to CD8 T cells [20]. NKG2D is constitutively expressed by all human CD8 T cells; however, it is expressed only by activated murine CD8 T cells [8]. Consistent with our previous report [28], targeting sMIC with B10G5 increased the frequency of NKG2D^+^ CD8 T cells in the dLN and in tumors (Fig. 5a and b). Although anti-PD-L1 mAb single agent only presented a marginal effect on NKG2D expression on CD8 T cells, combination therapy of anti-PD-L1 mAb and B10G5 resulted in an increase in the frequency of NKG2D^+^ CD8 T cells in draining LN and tumor infiltrates as compared to B10G5 single agent (Fig. 5a and b). These data suggest potential synergistic effects of B10G5 targeting MIC and anti-PD-L1 mAb in restoring and sustaining NKG2D expression on activated CD8 T cells.

Intriguingly, B10G5 therapy also resulted in a significant increase in the expression of CD28 on CD8 T cells in draining LN and tumors (Fig. 5c and d). No significant changes in the expression of CD28 or NKG2D was observed on CD4 T cells in tumor draining LN (Additional file 1: Figure S4). Anti-PD-L1 mAb therapy did not affect CD28 expression on CD8 T cells (Fig. 5c and d). Together, these data suggest that B10G5 enhances tumor response to anti-PD-L1 therapy in part by providing antigen-specific CD8 T cells with NKG2D and CD28 dual co-stimulation.

### Targeting sMIC provides enhanced and sustained NKG2D and CD28 dual co-stimulation to amplify TCR-mediated CD8 T cell activation

**Fig. 6:**
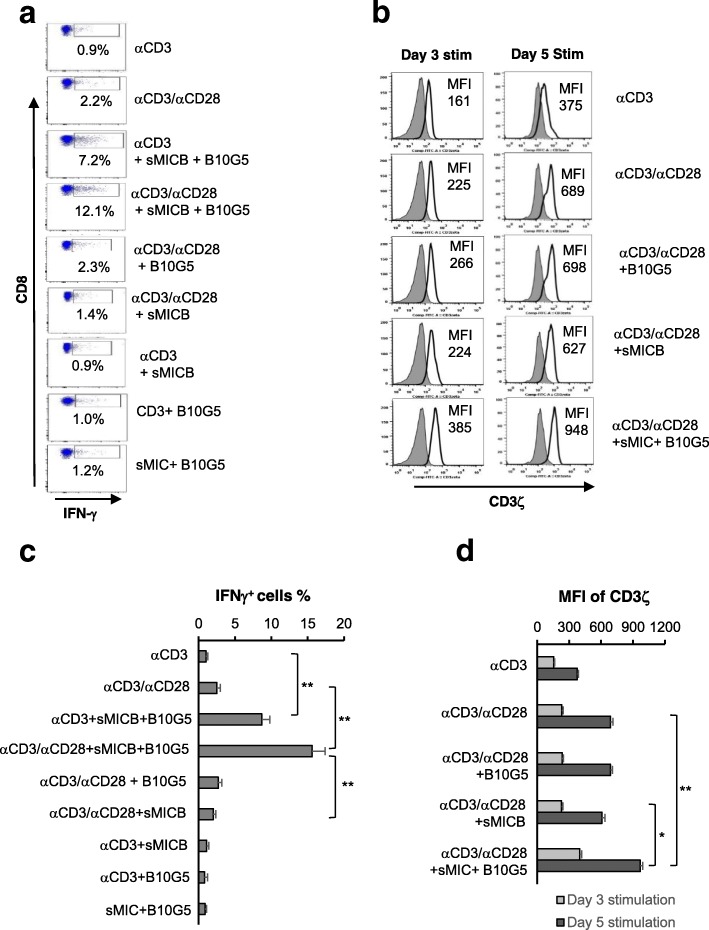
sMIC-targeting antibody and a CD28 agonist antibody provides CD8 TCR with dual co-stimulation and thus optimal and sustained activation. **a**, SV40TAg-specific TCR-I CD8 T cells were stimulated with the indicated conditions and evaluated for intracellular IFNγ production after 24 h of stimulation. **b**, Cells were stimulated with indicated conditions for three and 5 days. Expression of the TCR/CD3 signaling molecule CD3ζ was assessed by flow cytometry analyses with intracellular staining. **c** and **d**, Representative summary data of (**a**) and (**b**) respectively. Data represent results of triplicates of four independent experiments

Activation of NKG2D and CD28 can provide non-redundant co-stimulation to CD8 T cells [12, 35]. We thus sought to understand the significance of increased, or at least sustained, NKG2D and CD28 expression in TCR-mediated CD8 T cell function. We stimulated the SV40TAg-specific TCR-I CD8T cells with various conditions as indicated in Fig. 6. Addition of anti-CD28 agonist antibody resulted in modest CD3/TCR-mediated activation and proliferation as indicated by IFNγ production (Fig. 6a). Addition of recombinant sMIC plus the anti-sMIC antibody notably increased the magnitude of CD3/TCR-mediated activation. Intriguingly, anti-CD28 together with sMIC plus B10G5 remarkably amplified CD3/TCR-mediated activation (Fig. 6a and c), suggested a potential synergistic co-stimulation of TCR-dependent effector function of CD8 T cells. The enhanced CD3/TCR signaling by an CD28 agonist together with sMIC plus B10G5 co-stimulation was further substantiated with independent experiments in which the expression of the critical CD3/TCR signaling molecule CD3ζ was upregulated and sustained with prolonged dual co-stimulation (Fig. 6b and d).

We have previously shown, the antibody B10G5 does not block the interaction of sMIC and NKG2D. Instead, the complex formed by sMIC and B10G5 presented continuous binding to NKG2D (Additional file 1: Figure S5). We further confirmed that the complex formed by sMIC and B10G5 co-stimulates antigen-specific CD8 T cell effector function through NKG2D with the human tyrosinase-specific HLA-A2-restricted TIL13831 cells. Addition of the sMIC/B10G5 complex to the co-culture of TIL13831 and the artificial HLA-A2+ antigen presenting T2A2 cells significantly enhanced TIL13831 effector responses to tyrosinase peptide stimulation as measured by intracellular staining of IFNγ, TNFα, and CD107a; blocking NKG2D abolished the effect of sMIC/B10G5 complex (Additional file 1: Figure S6). Together, our data suggest a novel mechanism whereby co-targeting sMIC with PD1/PDL1 blockade therapy enhances antigen-specific CD8 effector T cell activation and tumor responses.

### Co-targeting sMIC with PD1/PD-L1 blockade increases NK cell number and function in the peripheral and tumors

**Fig. 7:**
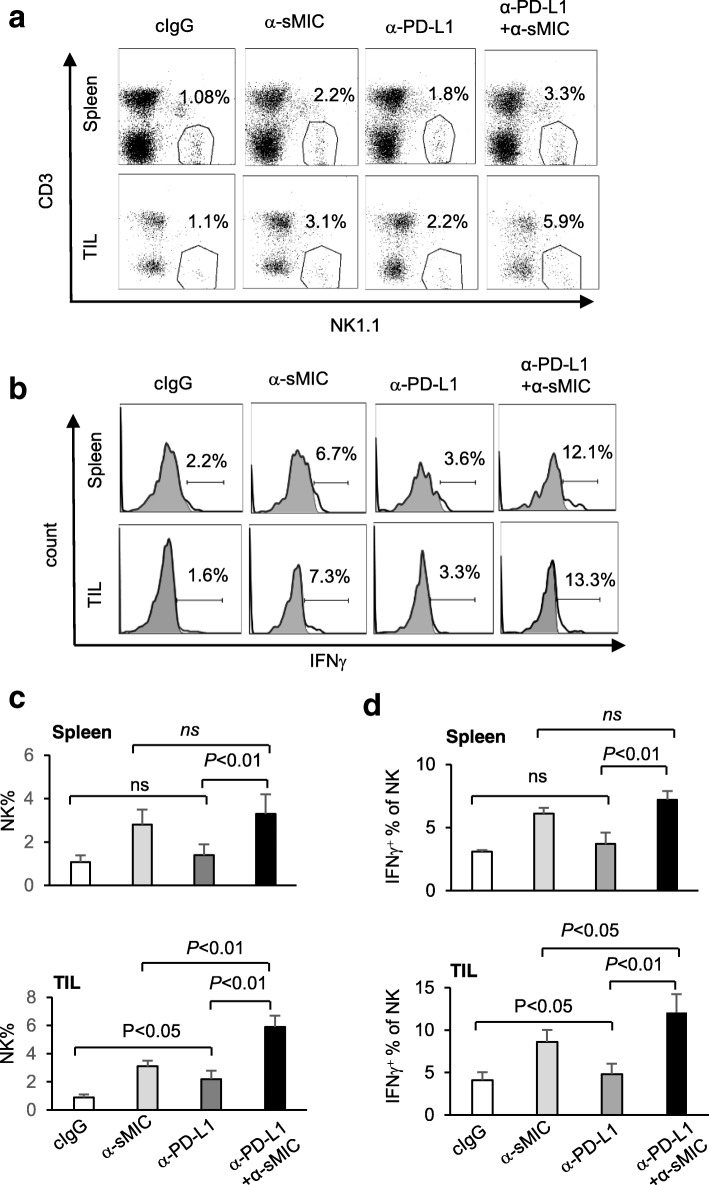
Antibody cocktail therapy of sMIC-targeting mAb B10G5 and anti-PD-L1 antibody cooperatively enriched NK cell infiltration and enhanced NK cell function in tumors. **a** and **b**, Representative dot plots (**a**) and summary data (**b**) from all animals present that antibody cocktail therapy significantly enriched NK cell in tumor infiltrates compared with monotherapy. **b** and **d**, Representative histograms (**b**) and summary data (**d**) from all animals present that antibody cocktail therapy significantly enhanced NK cell responsiveness in tumors compared with monotherapy. Note that anti-PD-L1 antibody monotherapy presented no significant effect on NK cells. TIL, tumor infiltrated lymphocytes. ns, not significant

As we have reported previously [28], targeting sMIC with B10G5 increased NK cell number in the peripheral and tumor infiltrates and augmented NK cell intrinsic function as measured by the ability to produce IFNγ in response to PMA and Ionomycin (PMA/I) stimulation (Fig. 7a–d). Anti-PD-L1 mAb monotherapy significantly increased NK cell numbers or intrinsic cellular function in tumors but not in the spleen (Fig. 7a–d). Combination therapy with B10G5 and the anti-PD-L1 mAb further significantly increased NK cell number and cellular intrinsic function only in tumors as compared to monotherapy of B10G5 or the anti-PD-L1 antibody (Fig. 7a–d). Notably, combination therapy significantly increased NKG2D expression on NK cells as compared to B10G5 single agent therapy, in spite that anti-PD-L1 mAb therapy alone did not impact NKG2D expression on NK cells (Additional file 1: Figure S7). The enhanced NKG2D expression may account in part for the functional enhancement of NK cells in response to the combination therapy. These observations suggest that NK cell may play a role in the synergistic effect of the anti-PD-L1 antibody and B10G5 targeting sMIC at tumor site.

### Targeting sMIC up-regulates PD-L1 expression on tumor cells which is in part NK-dependent

**Fig. 8:**
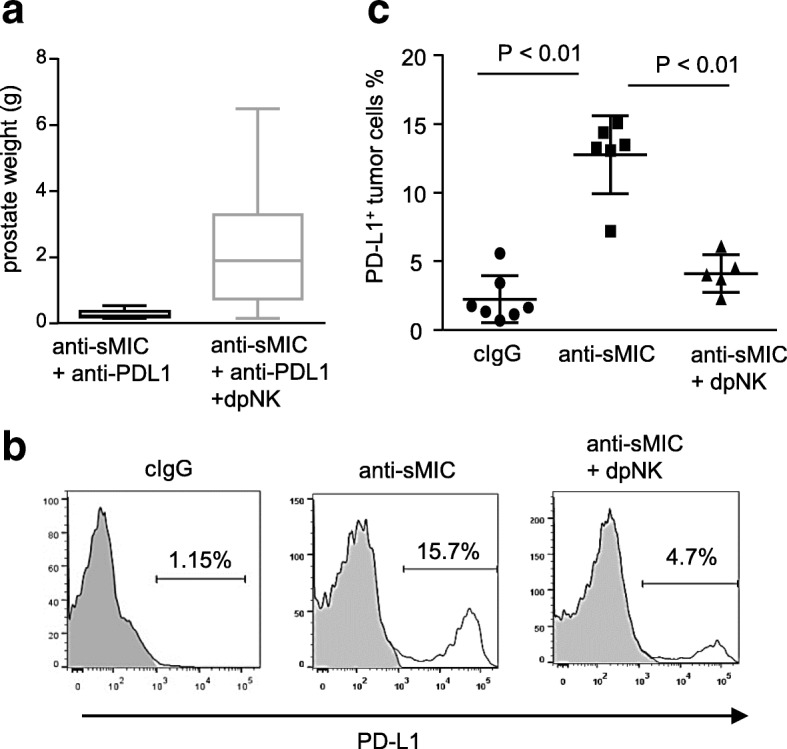
Anti-sMIC therapy with B10G5 up-regulates PD-L1 expression on tumor cells, which is in part NK cell dependent. **a**, Depletion of NK cells (dpNK) during therapy diminishes the therapeutic effect of co-targeting sMIC and PD-L1 as assessed by prostate weight at necropsy. All treatments were given twice weekly *i.p.* for 8 weeks. **b**, Representative histograms from flow cytometry analyses demonstrating the percentage of PD-L1^+^ tumor cells from TRAMP/MICB mice. **c**, Summary data of the percentage of PD-L1^+^ tumor cells from (**a**)

We have previously shown that depletion of NK cells compromises the therapeutic effect of B10G5 [28]. We thus investigated the impact of NK cell on the therapeutic efficacy of the combination therapy. Depletion NK cells significant impair the therapeutic outcome of the combination therapy of B10G5 and anti-PD-L1 in TRAMP/MICB mice as evaluated by prostate weight at necropsy (Fig. 8a).

We further investigated the impact of NK on enhancing the cooperative therapeutic effect of co-targeting sMIC and PD1/PD-L1 blockade. Lack of PD-L1 expression on tumor cells was thought at least in part contributing to the limited response to PD1/PD-L1 blockade therapy [37]. Interestingly, B10G5 anti-sMIC therapy significantly increased the percentage of tumor cells expressing PD-L1 (Fig. 8b). Depletion of NK cells during therapy diminished the upregulation of PD-L1 on tumor cells (Fig. 8b and c).Together, these data elucidate the significance impact of NK cell on the cooperative therapeutic efficacy of co-targeting sMIC and the PD1/PD-L1 pathway.

## Discussion

While immune checkpoint CTLA4 or PD-1 blockade therapy, monotherapy or combined, achieved significant survival benefits in patients with metastatic melanoma, a highly immunogenic human tumor [38–40]. For poorly immunogenic tumors where ongoing immune response is nominal, additional modalities, such as strong co-stimulatory signals to amplify the TCR-CD3 signaling, are needed to achieve the benefit of blocking co-inhibitory signals. Tumor-derived sMIC has been documented to impair NKG2D-mediated co-stimulation and CTL activation [20]. In our study, we presented that targeting sMIC with a non-blocking antibody provided CD8 T cells with dual co-stimulation. This augmented co-stimulation of CD8 T cells together with co-inhibition of the PD1/PD-L1 pathways enables optimal CD8 T cell activation in the environment of immune suppressive sMIC^+^.

High levels of circulating soluble NKG2D ligands have been associated with a poor clinical outcome in many solid tumors and poor response of PD1/PD-L1 blockade therapy as shown in melanoma patients [24–26, 30, 41, 42]. Our findings provide the pre-clinical proof-of-concept evidence that antibody targeting serum sMIC can enable and enhance sMIC^+^/MIC^+^ tumors to respond to PD1/PD-L1 blockade therapy. We demonstrate a cooperative therapeutic effect of an anti-PD-L1 mAb in combination with targeting sMIC. Our data demonstrate that the cooperatively enhanced therapeutic effect of the combination therapy is associated with markedly enhance antigen-enhanced CD8 T cell function and sustainability in tumors, augmented DC functional potential by increasing the expression of co-stimulatory molecules CD80/86 and CD40, and enriched NK cell in tumors with enhanced anti-tumor effector function. We previously showed that targeting sMIC with B10G5 mAb invigorates NK and CD8 T cell anti-tumor immunity and augments DC costimulatory potential [43] [28]. Here, we show that targeting sMIC in combination with anti-PD-L1 antibody further enhances the expression of CD80 and CD86 on DCs in draining LN and tumors as compared to given single agent therapy, suggesting an increased ability of DCs to prime antigen-specific CD8 T cells. Intriguingly, our data show that CD28 expression on CD8 T cells in draining LN and tumors was upregulated with B10G5 targeting sMIC. Together with increased CD80/86 on DCs with combined therapy, it is anticipated to instrument CD8 T cells with a strong co-stimulation signal for priming. Our data also demonstrate that B10G5 stabilizes the expression of NKG2D which provides direct co-simulation of CD8 T cells by MIC-positive tumor cells. As CD28 and NKG2D provide non-redundant co-stimulatory signals [12, 36], NKG2D stabilization and CD28 upregulation would provide dual-co-stimulation to antigen-specific CD8 T cells. The enhanced co-stimulation together with blocking PD1/PD-L1 inhibitory is instrumented to provide optimal amplification of CD3/TCR signaling and activation of antigen-specific CD8 T cells as demonstrated in our antigen-specific TCR-I CD8 adoptive transfer studies. This enhanced co-stimulation is critical particularly for poorly immunogenic tumors, where ongoing immune response is nominal, to achieve the benefit of blocking PD1/PD-L1 inhibitory signals.

Tumor-derived sMIC was shown to induce degradation of CD3ζ in CD8 T cells and NK cells [23]. The ITAM-containing CD3ζ molecule is critical for TCR signaling [44]. Phosphorylation of the ITAMs on the CD3ζ by the Src-family kinase Lck is the initial step that initiates downstream signaling from TCR upon antigen presentation [45, 46]. Thus, sustained expression or the stability of CD3ζ is instrumental to maintain antigen-specific CD8 T cell function. We have previously shown that targeting sMIC with B10G5 neutralizes the negative impact of sMIC and stabilizes CD3ζ expression [31]. We show in this study that sMIC-targeting together with CD28 co-stimulation remarkably increase and sustain CD3ζ expression in CD8 T cell even with prolonged TCR/CD3 complex engagement. This increased stability of CD3ζ is highly relevant to the amplification of TCR signaling and functional readout of IFNγ production as our data have shown.

Our data show that co-stimulation to CD8 T cells by CD28 and NKG2D through sMIC/B10G5 generated a higher magnitude of amplifying CD3/TCR signaling than each individual component. It has been shown that CD28 and NKG2D signaling may provide different necessity to for human naïve and effector CD8 T cells [11, 12, 47], despite that the two co-stimulatory pathways share certain signaling components, such as Grb2 and p85 subunit of PI3K [35]. Simultaneous signals from the TCR complex and NKG2D was shown to be able alter CD28 co-stimulatory signal transduction pathways in human CD8^+^ T cells [47]. Recent study by Prez et al. showed that NKG2D signaling in CD8 T cells is necessary during the effector phase for the development of functional memory cells [20]. Together, these studies support the notion that NKG2D and CD28 provide non-redundant activation signal to support TCR-dependent CD8 T cell function. The question how targeting sMIC together with CD28 co-stimulation would determine the fate of memory CD8 T cell differentiation after initial activation warrants further investigation.

Our data show that co-targeting sMIC with PD1/PD-L1 blockade therapy significantly increased NK population in the peripheral and tumors and improved NK cell intrinsic functional competency. We have shown in earlier studies that sMIC significantly disturbs NK cell peripheral maintenance and function in cancer patients and pre-clinical models and that targeting sMIC rescues these defects on NK cells [21, 27, 28]. How combination with PD1/PD-L1 blockade cooperatively enhance NK cell number and function could be a result of multiple pathways. PD1/PD-L1 blockade could have direct impact in enhancing NK cell function. It has been shown anti-PD1 antibody improves NK cell function in multiple myeloma patients through direct disrupting PD-1 signaling on NK cells [48]. Our data show that the combination therapy significantly augmented DC functional potential. Given the crosstalk between DC and NK cells, an indirect impact on NK cells through this pathway is also anticipated. High numbers of circulating pool of functionally competent NK cells at baseline or during treatment associate with better clinical outcome of anti-PD1 treatment in advanced NSCLC [49]. These studies imply a significant interplay, directly or indirectly, between NK cell function and PD1/PD-L1 blockade.

Interestingly, we show that PD-L1 expression in tumor cells is highly related to sMIC level in serum, presumably reflecting sMIC level at the tumor site(s). One of the major immune suppressive effect of sMIC is to impair NK cell immunity [16, 27, 50]. Given that NK cells are the major source of IFNγ, a key regulator of PD-L1 expression [51–53], consequently, impaired NK cell function by sMIC would anticipate to negatively impact PD-L1 expression on tumors and potentially other cell types. Our findings provided at least one aspect of mechanistic understanding the clinical observation that patients with high levels of soluble NKG2D ligands elicited poor response to PD1/PD-L1 blockade therapy [26, 30]. Tissue levels of PD-L1 expression is a mandatory biomarker for patient population selection to receive anti-PD-L1 therapy in a number of cancer types where tissue biopsy is achievable. In cancer types, such as metastatic prostate cancer, where obtaining metastatic tissue biopsy is challenging, our study suggests that serum levels of sMIC may serve as an alternative biomarker to exclude patients who are not likely respond to PD1/PD-L1 blockade treatment. Clinical study to validate our observations in the TRAMP/MIC animals is warranted.

Noteworthy, although our study was mostly performed with the TRAMP/MIC prostate tumor model, as we have described, TRAMP/MIC tumor shedding of MIC is associated with tumor progression and metastasis. This biology resembles the MIC-NKG2D mediated oncoimmunology biology in a broad-spectrum MIC^+^ cancer patients [24–26]. Thus, the biology warrants this proof-of-concept therapeutic potential to be applicable to a broad range of cancer patients with high levels of circulating sMIC^+^.

## Conclusion

Our data presented a previously uncharacterized immune mechanism by which the antibody B10G5 targeting sMIC enhances the efficacy of PD1/PD-L1blockade against sMIC^+^ tumors. Mechanistically, co-targeting sMIC optimally activates antigen-specific CD8 T cells by providing NKG2D- and CD28-mediated dual co-stimulation in addition to blocking the PD1/PD-L1 inhibitory pathways. Our data also presented that co-targeting sMIC during PD1/PD-L1 blockade therapy augments DC antigen-presenting potential and NK cell anti-tumor competency. This study provides a mechanism-driven proof-of-concept evidence to support a novel combination therapy for treating the PD1/PD-L1 poor responders that are positive for sMIC.

## Additional file

Additional file 1: **Figure S1** Serum levels of sMICB in animal cohorts at pre-treatment baseline. **Figure S2** sMIC expressing tumor cells elicited impaired response to anti-PD-L1 therapy. **Figure S3** Cooperative therapy effect of anti-PD1 mAb and sMIC-targeting mAb B10G5. **Figure S4** clearance of sMIC does not increase co-stimulatory molecules CD28 or NKG2D on CD4 T cells in tumor draining lymph node (dLN). **Figure S5** Detection of sMIC (A and B) binds to NKG2D and B10G5 simultaneously. **Figure S6** sMIC/B10G5 co-stimulation amplifies antigen-specific TCR-signaling through NKG2D. **Figure S7** Therapy results in increase in NKG2D expression on NK cells. (PPTX 2068 kb)

## Data Availability

The datasets used and/or analyzed during the current study are available. from the corresponding author on reasonable request.

## Abbreviations

DC: Dendritic cells
MIC: MHC I chain related molecule
NK: Natural killer
sMIC: Soluble MIC
TCR: T cell receptor
TRAMP: Transgenic Adenocarcinoma of *Mouse* Prostate
ULBP: UL-16 binding protein

## References

[1] Acuto O, Michel F (2003). CD28-mediated co-stimulation: a quantitative support for TCR signalling. Nat Rev Immunol.

[2] Prlic M, Williams MA, Bevan MJ (2007). Requirements for CD8 T-cell priming, memory generation and maintenance. Curr Opin Immunol.

[3] Williams MA, Bevan MJ (2007). Effector and memory CTL differentiation. Annu Rev Immunol.

[4] Sanmamed MF (2015). Agonists of co-stimulation in Cancer immunotherapy directed against CD137, OX40, GITR, CD27, CD28, and ICOS. Semin Oncol.

[5] Croft M (2009). The significance of OX40 and OX40L to T-cell biology and immune disease. Immunol Rev.

[6] Bauer S (1999). Activation of NK cells and T cells by NKG2D, a receptor for stress-inducible MICA. Science.

[7] Jamieson AM (2002). The role of the NKG2D immunoreceptor in immune cell activation and natural killing. Immunity.

[8] Raulet DH (2003). Roles of the NKG2D immunoreceptor and its ligands. Nat Rev Immunol.

[9] Roberts AI (2001). NKG2D receptors induced by IL-15 costimulate CD28-negative effector CTL in the tissue microenvironment. J Immunol.

[10] Zhang J, Basher F, Wu JD (2015). NKG2D Ligands in Tumor Immunity: Two Sides of a Coin. Front Immunol.

[11] Groh V (2001). Costimulation of CD8alphabeta T cells by NKG2D via engagement by MIC induced on virus-infected cells. Nat Immunol.

[12] Rajasekaran Kamalakannan, Xiong Va, Fong Lee, Gorski Jack, Malarkannan Subramaniam (2010). Functional Dichotomy between NKG2D and CD28-Mediated Co-Stimulation in Human CD8+ T Cells. PLoS ONE.

[13] Groh V (1999). Broad tumor-associated expression and recognition by tumor-derived gamma delta T cells of MICA and MICB. Proc Natl Acad Sci U S A.

[14] Bahram S, Spies T (1996). The MIC gene family. Res Immunol.

[15] Kasahara M, Sutoh Y (2015). Comparative genomics of the NKG2D ligand gene family. Immunol Rev.

[16] Maurer S (2018). Platelet-mediated shedding of NKG2D ligands impairs NK cell immune-surveillance of tumor cells. Oncoimmunology.

[17] Salih HR, Holdenrieder S, Steinle A (2008). Soluble NKG2D ligands: prevalence, release, and functional impact. Front Biosci.

[18] Baragano Raneros A, Suarez-Alvarez B, Lopez-Larrea C (2014). Secretory pathways generating immunosuppressive NKG2D ligands: new targets for therapeutic intervention. Oncoimmunology.

[19] Chitadze G (2013). Shedding of endogenous MHC class I-related chain molecules a and B from different human tumor entities: heterogeneous involvement of the “a disintegrin and metalloproteases” 10 and 17. Int J Cancer.

[20] Groh V (2002). Tumour-derived soluble MIC ligands impair expression of NKG2D and T-cell activation. Nature.

[21] Wu JD (2004). Prevalent expression of the immunostimulatory MHC class I chain-related molecule is counteracted by shedding in prostate cancer. J Clin Invest.

[22] Xiao G (2015). Soluble NKG2D ligand promotes MDSC expansion and skews macrophage to the alternatively activated phenotype. J Hematol Oncol.

[23] Hanaoka N (2010). NKG2D initiates caspase-mediated CD3zeta degradation and lymphocyte receptor impairments associated with human cancer and autoimmune disease. J Immunol.

[24] Holdenrieder S (2006). Soluble MICB in malignant diseases: analysis of diagnostic significance and correlation with soluble MICA. Cancer Immunol Immunother.

[25] Holdenrieder S (2006). Soluble MICA in malignant diseases. Int J Cancer.

[26] Lopez-Soto A, Gonzalez S, Galluzzi L (2017). Soluble NKG2D ligands limit the efficacy of immune checkpoint blockade. Oncoimmunology.

[27] Liu G (2013). Perturbation of NK cell peripheral homeostasis accelerates prostate carcinoma metastasis. J Clin Invest.

[28] Lu S, et al. Non-blocking monoclonal antibody targeting soluble MIC revamps endogenous innate and adaptive anti-tumor responses and eliminates primary and metastatic tumors. Clin Cancer Res. 2015;21(21):4819-30. 10.1158/1078-0432.CCR-15-0845 PMC4631684 26106076

[29] Staveley-O'Carroll K (2003). In vivo ligation of CD40 enhances priming against the endogenous tumor antigen and promotes CD8+ T cell effector function in SV40 T antigen transgenic mice. J Immunol.

[30] Maccalli C (2017). Soluble NKG2D ligands are biomarkers associated with the clinical outcome to immune checkpoint blockade therapy of metastatic melanoma patients. Oncoimmunology.

[31] Zhang J (2017). Antibody-mediated neutralization of soluble MIC significantly enhances CTLA4 blockade therapy. Sci Adv.

[32] Wu JD (2009). Obstructing shedding of the immunostimulatory MHC class I chain-related gene B prevents tumor formation. Clin Cancer Res.

[33] Parisotto M, Metzger D (2013). Genetically engineered mouse models of prostate cancer. Mol Oncol.

[34] Bai A (2008). Rapid tolerization of virus-activated tumor-specific CD8+ T cells in prostate tumors of TRAMP mice. Proc Natl Acad Sci U S A.

[35] Prajapati K (2018). Functions of NKG2D in CD8(+) T cells: an opportunity for immunotherapy. Cell Mol Immunol.

[36] Markiewicz MA (2005). Costimulation through NKG2D enhances murine CD8+ CTL function: similarities and differences between NKG2D and CD28 costimulation. J Immunol.

[37] Hansen AR (2018). Pembrolizumab for advanced prostate adenocarcinoma: findings of the KEYNOTE-028 study. Ann Oncol.

[38] Hodi FS (2010). Improved survival with ipilimumab in patients with metastatic melanoma. N Engl J Med.

[39] Larkin J, Hodi FS, Wolchok JD (2015). Combined Nivolumab and Ipilimumab or monotherapy in untreated melanoma. N Engl J Med.

[40] Wolchok JD (2015). PD-1 Blockers. Cell.

[41] Tamaki S (2010). Soluble MICB serum levels correlate with disease stage and survival rate in patients with oral squamous cell carcinoma. Anticancer Res.

[42] Zhao YK (2015). Expression and clinical value of the soluble major histocompatibility complex class I-related chain a molecule in the serum of patients with renal tumors. Genet Mol Res.

[43] Wu J (2016). Antibody targeting soluble NKG2D ligand sMIC refuels and invigorates the endogenous immune system to fight cancer. Oncoimmunology.

[44] Birnbaum ME (2014). Molecular architecture of the alphabeta T cell receptor-CD3 complex. Proc Natl Acad Sci U S A.

[45] Shah NH, et al. An electrostatic selection mechanism controls sequential kinase signaling downstream of the T cell receptor. Elife. 2016;5:e20105. 10.7554/eLife.20105 PMC5089863 27700984

[46] Smith-Garvin JE, Koretzky GA, Jordan MS (2009). T cell activation. Annu Rev Immunol.

[47] Barber A, Sentman CL (2011). NKG2D receptor regulates human effector T-cell cytokine production. Blood.

[48] Benson DM (2010). The PD-1/PD-L1 axis modulates the natural killer cell versus multiple myeloma effect: a therapeutic target for CT-011, a novel monoclonal anti-PD-1 antibody. Blood.

[49] Mazzaschi G (2019). The circulating pool of functionally competent NK and CD8+ cells predicts the outcome of anti-PD1 treatment in advanced NSCLC. Lung Cancer.

[50] Kloss S (2015). Increased sMICA and TGFbeta1 levels in HNSCC patients impair NKG2D-dependent functionality of activated NK cells. Oncoimmunology.

[51] Alspach E, Lussier DM, Schreiber RD. Interferon gamma and its important roles in promoting and inhibiting spontaneous and therapeutic Cancer immunity. Cold Spring Harb Perspect Biol. 2019;11:a028480. 10.1101/cshperspect.a028480 PMC6396335 29661791

[52] Garcia-Diaz A (2017). Interferon receptor signaling pathways regulating PD-L1 and PD-L2 expression. Cell Rep.

[53] Osum KC (2018). Interferon-gamma drives programmed death-ligand 1 expression on islet beta cells to limit T cell function during autoimmune diabetes. Sci Rep.

