# Supplementary material for: Antibody targeting tumor-derived soluble NKG2D ligand sMIC provides dual co-stimulation of CD8 T cells and enables sMIC+ tumors respond to PD1/PD-L1 blockade therapy
Source: J Immunother Cancer. 2019 Aug 26;7:223. doi: 10.1186/s40425-019-0693-y (PMC6709558; doi:10.1186/s40425-019-0693-y)
Supplement: Supplementary file 1 — Figure S1 Serum levels of sMICB in animal cohorts at pre-treatment baseline. Figure S2 sMIC expressing tumor cells elicited impaired response to anti-PD-L1 therapy. Figure S3 Cooperative therapy effect of anti-PD1 mAb and sMIC-targeting mAb B10G5. Figure S4 clearance of sMIC does not increase co-stimulatory molecules CD28 or NKG2D on CD4 T cells in tumor draining lymph node (dLN). Figure S5 Detection of sMIC (A and B) binds to NKG2D and B10G5 simultaneously. Figure S6 sMIC/B10G5 co-stimulation amplifies antigen-specific TCR-signaling through NKG2D. Figure S7 Therapy results in increase in NKG2D expression on NK cells. (PPTX 2068 kb) [file 40425_2019_693_MOESM1_ESM.pptx]

## Slide 1
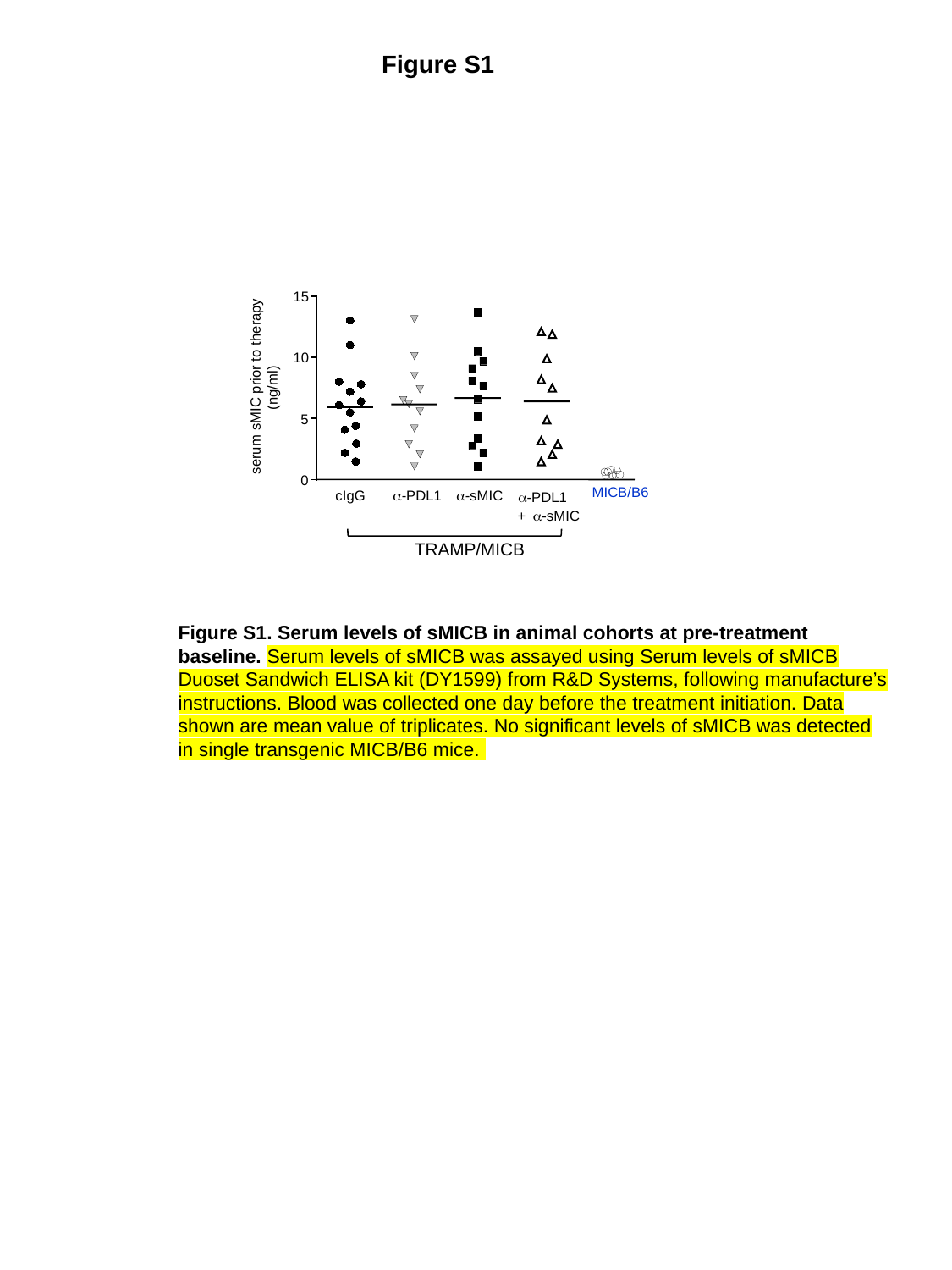

Figure S1
15
10
5
0
MICB/B6
cIgG
a-PDL1
a-sMIC
a-PDL1
+ a-sMIC
TRAMP/MICB
serum sMIC prior to therapy
 (ng/ml)
Figure S1. Serum levels of sMICB in animal cohorts at pre-treatment baseline. Serum levels of sMICB was assayed using Serum levels of sMICB
Duoset Sandwich ELISA kit (DY1599) from R&D Systems, following manufacture’s instructions. Blood was collected one day before the treatment initiation. Data shown are mean value of triplicates. No significant levels of sMICB was detected in single transgenic MICB/B6 mice.

## Slide 2
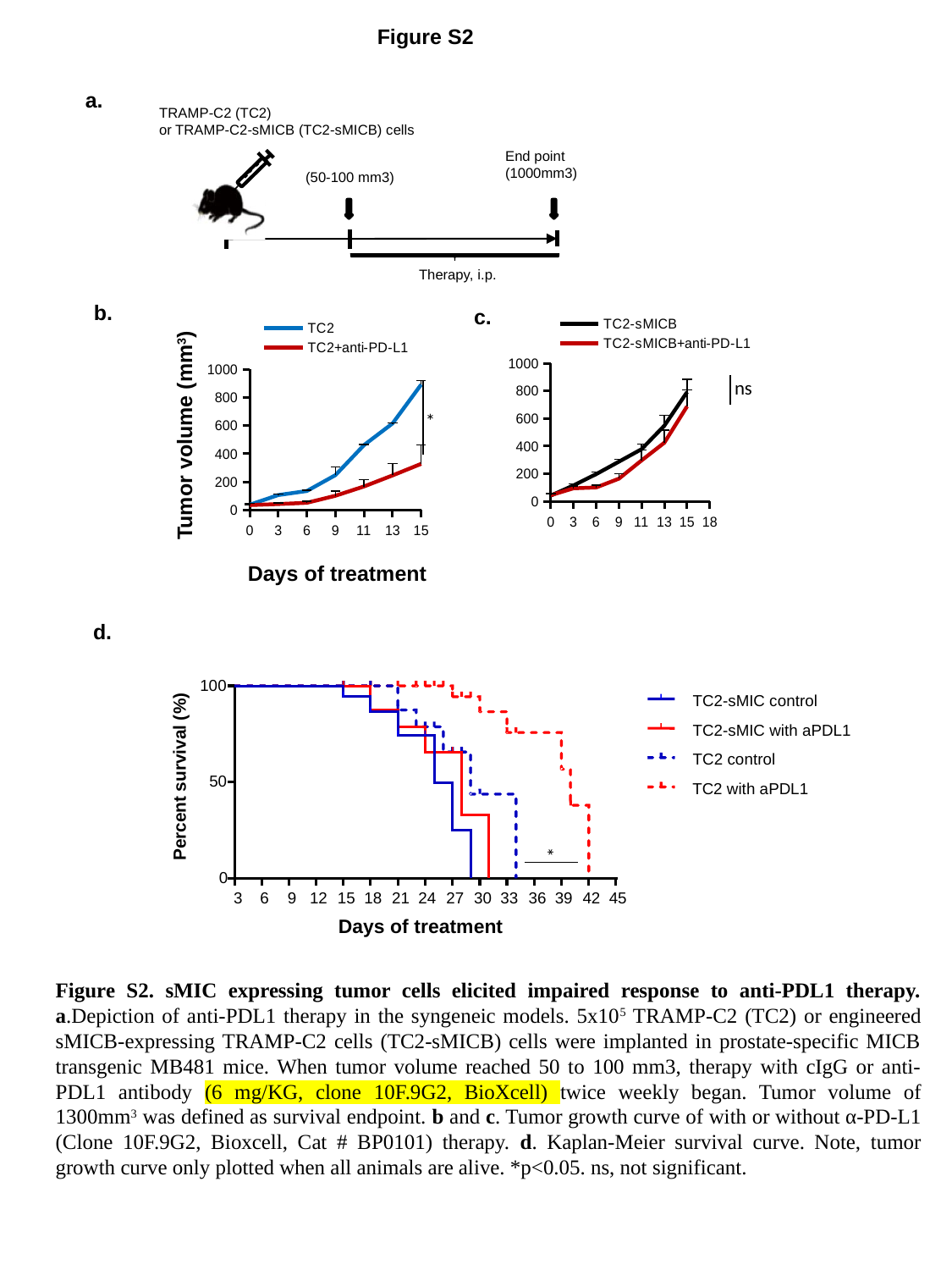

Figure S2
a.
TRAMP-C2 (TC2)
or TRAMP-C2-sMICB (TC2-sMICB) cells
End point
(1000mm3)
(50-100 mm3)
Therapy, i.p.
b.
c.
[unsupported chart]
### Chart
| Category | | |
|---|---|---|
| 0 | 37.583749999999995 | 34.67333333333333 |
| 3 | 106.02000000000001 | 42.0295 |
| 6 | 134.88875000000002 | 50.834333333333326 |
| 9 | 247.584 | 100.85066666666667 |
| 11 | 460.72125000000005 | 167.18583333333333 |
| 13 | 614.881 | 245.36266666666666 |
| 15 | 890.6890000000001 | 327.59700000000004 |ns
*
Tumor volume (mm3)
Days of treatment
d.
100
50
0
3
6
9
12
15
18
21
24
27
30
33
36
39
42
45
TC2-sMIC control
TC2-sMIC with aPDL1
Percent survival (%)
TC2 control
TC2 with aPDL1
*
Days of treatment
Figure S2. sMIC expressing tumor cells elicited impaired response to anti-PDL1 therapy. a.Depiction of anti-PDL1 therapy in the syngeneic models. 5x10^5^ TRAMP-C2 (TC2) or engineered sMICB-expressing TRAMP-C2 cells (TC2-sMICB) cells were implanted in prostate-specific MICB transgenic MB481 mice. When tumor volume reached 50 to 100 mm3, therapy with cIgG or anti-PDL1 antibody (6 mg/KG, clone 10F.9G2, BioXcell) twice weekly began. Tumor volume of 1300mm3 was defined as survival endpoint. b and c. Tumor growth curve of with or without α-PD-L1 (Clone 10F.9G2, Bioxcell, Cat # BP0101) therapy. d. Kaplan-Meier survival curve. Note, tumor growth curve only plotted when all animals are alive. *p<0.05. ns, not significant.

## Slide 3
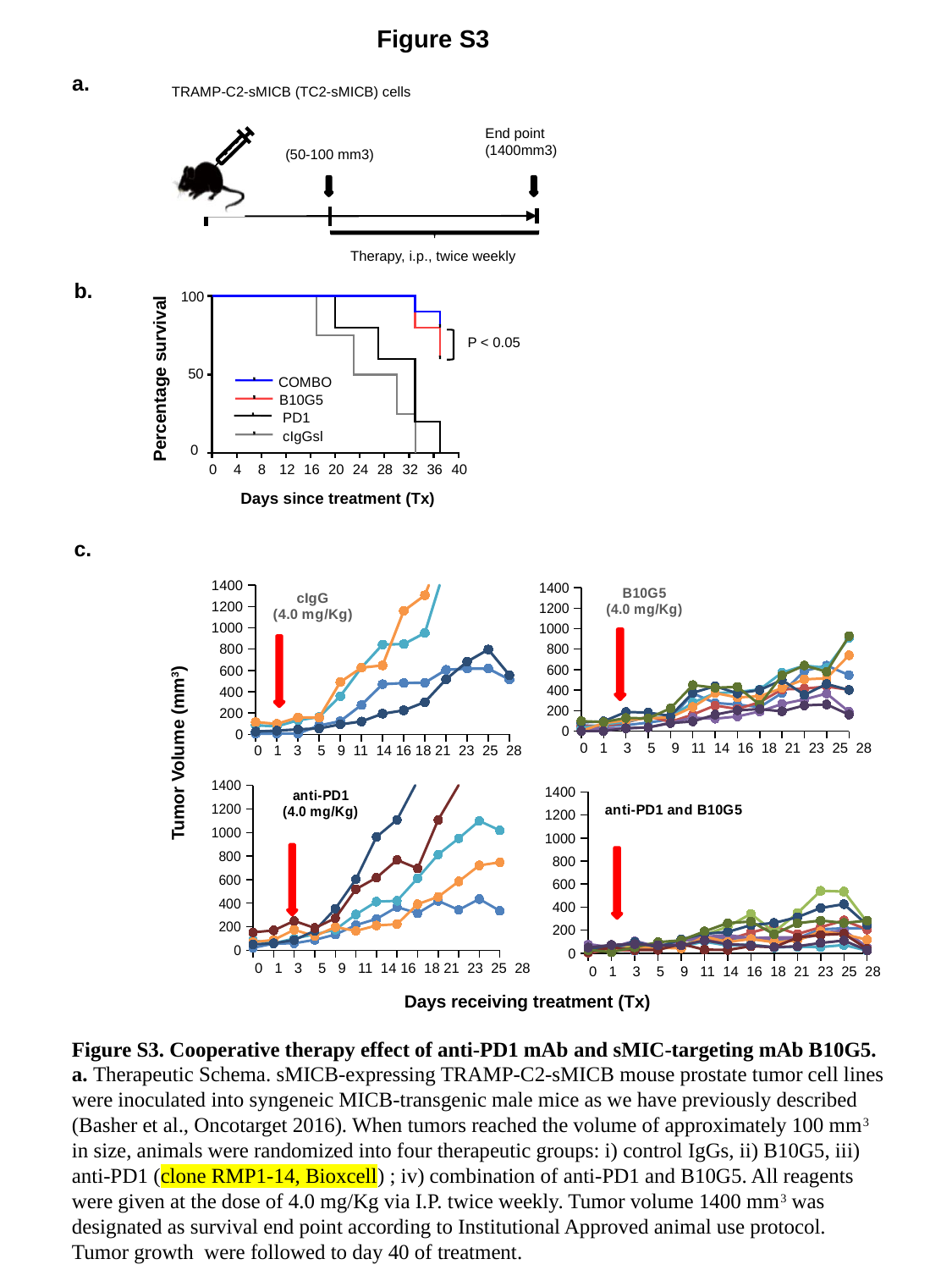

Figure S3
a.
TRAMP-C2-sMICB (TC2-sMICB) cells
End point
(1400mm3)
(50-100 mm3)
Therapy, i.p., twice weekly
b.
100
COMBO
B10G5
PD1
cIgGsl
0
4
8
12
16
20
24
28
32
36
40
Days since treatment (Tx)
P < 0.05
Percentage survival
50
0
c.
### Chart: B10G5
(4.0 mg/Kg)
| Category | | | | | | | | |
|---|---|---|---|---|---|---|---|---|
| 22 | 55.47 | 48.4 | 24.025 | 28.512 | 0.0 | 85.2355 | 97.2 | 0.0 |
| 23 | 49.392 | 48.51 | 26.112 | 63.626 | 77.2475 | 94.9365 | 87.88 | 0.0 |
| 25 | 57.33 | 83.75 | 26.112 | 92.2625 | 105.3375 | 186.368 | 127.8 | 27.864 |
| 27 | 82.524 | 141.5375 | 35.2 | 135.168 | 124.6535 | 180.5859 | 124.2 | 36.011 |
| 31 | 114.0475 | 89.888 | 81.312 | 134.54 | 134.54 | 147.392 | 222.376 | 77.9625 |
| 33 | 368.082 | 161.172 | 131.0 | 278.4375 | 235.2 | 374.166 | 449.0 | 94.1625 |
| 36 | 275.128 | 249.5 | 120.9 | 369.82 | 371.3 | 437.3 | 423.0 | 160.1 |
| 38 | 257.725 | 215.8245 | 142.884 | 380.701 | 328.536 | 368.082 | 431.664 | 202.8 |
| 40 | 234.423 | 281.6 | 191.455 | 406.8 | 340.6 | 400.221 | 258.11 | 214.245 |
| 43 | 371.25 | 406.53 | 264.23 | 571.32 | 419.813 | 499.488 | 543.95 | 192.6 |
| 45 | 586.625 | 414.05 | 309.0 | 640.0 | 505.0 | 352.87 | 640.45 | 250.36 |
| 47 | 640.0 | 435.22 | 366.1 | 617.1 | 515.15 | 459.27 | 580.064 | 259.83 |
| 50 | 546.5 | 402.17 | 189.06 | 908.89 | 740.602 | 400.7595 | 928.0 | 160.38 |
### Chart: cIgG
(4.0 mg/Kg)
| Category | | | | |
|---|---|---|---|---|
| 22 | 6.877 | 86.098 | 115.3359 | 26.95 |
| 23 | 8.25 | 75.429 | 98.696 | 35.301 |
| 25 | 8.0 | 132.9615 | 159.744 | 47.5875 |
| 27 | 83.125 | 163.35 | 157.604 | 56.448 |
| 31 | 123.92 | 358.9 | 490.398 | 94.08 |
| 33 | 275.684 | 625.931 | 625.864 | 120.9325 |
| 36 | 470.59 | 843.55 | 647.3 | 194.5 |
| 38 | 481.526 | 847.226 | 1158.948 | 225.264 |
| 40 | 485.002 | 951.28 | 1304.688 | 303.0 |
| 43 | 605.696 | 1593.983 | 1813.0 | 516.096 |
| 45 | 617.16 | 1800.0 | 1813.0 | 682.344 |
| 47 | 617.463 | None | None | 796.54 |
| 50 | 514.0 | None | None | 555.9 |0 1 3 5 9 11 14 16 18 21 23 25 28
0 1 3 5 9 11 14 16 18 21 23 25 28
Tumor Volume (mm3)
### Chart: anti-PD1
(4.0 mg/Kg)
| Category | | | | | |
|---|---|---|---|---|---|
| 22 | 21.168 | 84.7 | 69.12 | 49.392 | 152.626 |
| 23 | 57.112 | 59.048 | 88.48 | 59.643 | 169.016 |
| 25 | 59.643 | 99.7195 | 173.95 | 87.079 | 249.4 |
| 27 | 89.056 | 134.946 | 120.0945 | 165.528 | 189.486 |
| 31 | 133.956 | 175.173 | 197.173 | 352.87 | 272.0 |
| 33 | 214.0815 | 304.0 | 164.7135 | 603.28 | 518.616 |
| 36 | 266.24 | 413.0 | 210.49 | 962.0 | 616.512 |
| 38 | 367.24 | 420.229 | 222.187 | 1108.02 | 766.584 |
| 40 | 314.2 | 611.8 | 391.072 | 1437.5 | 695.3955 |
| 43 | 419.2 | 813.186 | 454.07 | 1638.876 | 1106.95 |
| 45 | 342.7 | 950.0 | 585.0 | None | 1402.63 |
| 47 | 435.2 | 1098.3 | 720.77 | None | None |
| 50 | 335.38 | 1017.55 | 746.98 | None | None |
### Chart: anti-PD1 and B10G5
| Category | | | | | | | | | | |
|---|---|---|---|---|---|---|---|---|---|---|
| 22 | 24.505 | 5.0 | 40.344 | 73.568 | 17.1 | 38.808 | 45.5625 | 9.375 | 28.175 | 48.749 |
| 23 | 27.864 | 26.864 | 26.95 | 43.56 | 51.304 | 31.23 | 45.496 | 49.953 | 9.375 | 71.79 |
| 25 | 21.4375 | 25.272 | 78.732 | 101.124 | 31.752 | 50.336 | 97.216 | 27.864 | 50.8475 | 78.732 |
| 27 | 78.336 | 53.958 | 77.064 | 48.668 | 42.4 | 46.575 | 50.625 | 27.864 | 95.648 | 68.9265 |
| 31 | 47.432 | 73.008 | 51.304 | 111.392 | 58.53 | 43.218 | 120.0945 | 80.0526 | 111.328 | 66.3255 |
| 33 | 115.351 | 100.602 | 158.994 | 135.594 | 100.62 | 143.748 | 167.936 | 30.456 | 188.65 | 111.012 |
| 36 | 121.8 | 116.03 | 229.1 | 151.838 | 66.15 | 97.216 | 184.049 | 27.56 | 261.6 | 78.416 |
| 38 | 141.53 | 178.955 | 340.736 | 123.87 | 76.032 | 122.79 | 243.089 | 60.75 | 274.6 | 68.923 |
| 40 | 100.7 | 230.625 | 183.7 | 132.09 | 48.8 | 95.28 | 262.4 | 52.0 | 164.25 | 52.0 |
| 43 | 128.99 | 166.09 | 346.8 | 129.96 | 56.376 | 109.33 | 312.132 | 135.759 | 259.6115 | 59.643 |
| 45 | 207.3 | 225.5 | 540.65 | 167.4 | 52.9 | 189.0 | 391.988 | 158.14 | 283.03 | 88.9 |
| 47 | 216.0 | 284.592 | 534.52 | 192.49 | 70.875 | 164.25 | 425.27 | 168.68 | 262.8 | 109.35 |
| 50 | 216.302 | 204.72 | 269.7695 | 45.486 | 22.23 | 116.6135 | 245.134 | 36.4815 | 281.6275 | 25.1125 |0 1 3 5 9 11 14 16 18 21 23 25 28
0 1 3 5 9 11 14 16 18 21 23 25 28
Days receiving treatment (Tx)
Figure S3. Cooperative therapy effect of anti-PD1 mAb and sMIC-targeting mAb B10G5.
a. Therapeutic Schema. sMICB-expressing TRAMP-C2-sMICB mouse prostate tumor cell lines were inoculated into syngeneic MICB-transgenic male mice as we have previously described (Basher et al., Oncotarget 2016). When tumors reached the volume of approximately 100 mm3 in size, animals were randomized into four therapeutic groups: i) control IgGs, ii) B10G5, iii) anti-PD1 (clone RMP1-14, Bioxcell) ; iv) combination of anti-PD1 and B10G5. All reagents were given at the dose of 4.0 mg/Kg via I.P. twice weekly. Tumor volume 1400 mm3 was designated as survival end point according to Institutional Approved animal use protocol. Tumor growth were followed to day 40 of treatment.

## Slide 4
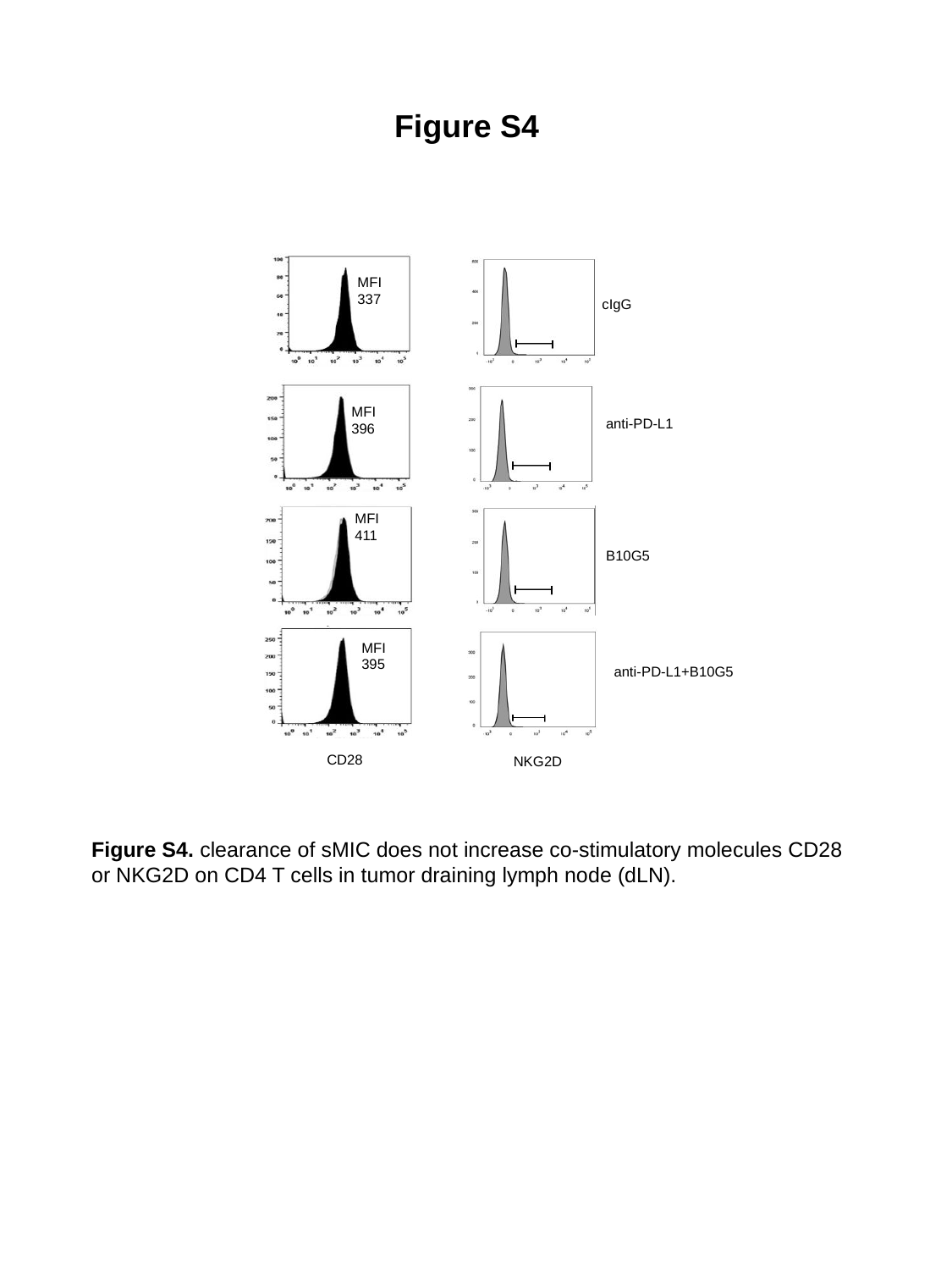

Figure S4
MFI
337
MFI
396
MFI
411
MFI
395
CD28
NKG2D
cIgG
anti-PD-L1
B10G5
anti-PD-L1+B10G5
Figure S4. clearance of sMIC does not increase co-stimulatory molecules CD28 or NKG2D on CD4 T cells in tumor draining lymph node (dLN).

## Slide 5
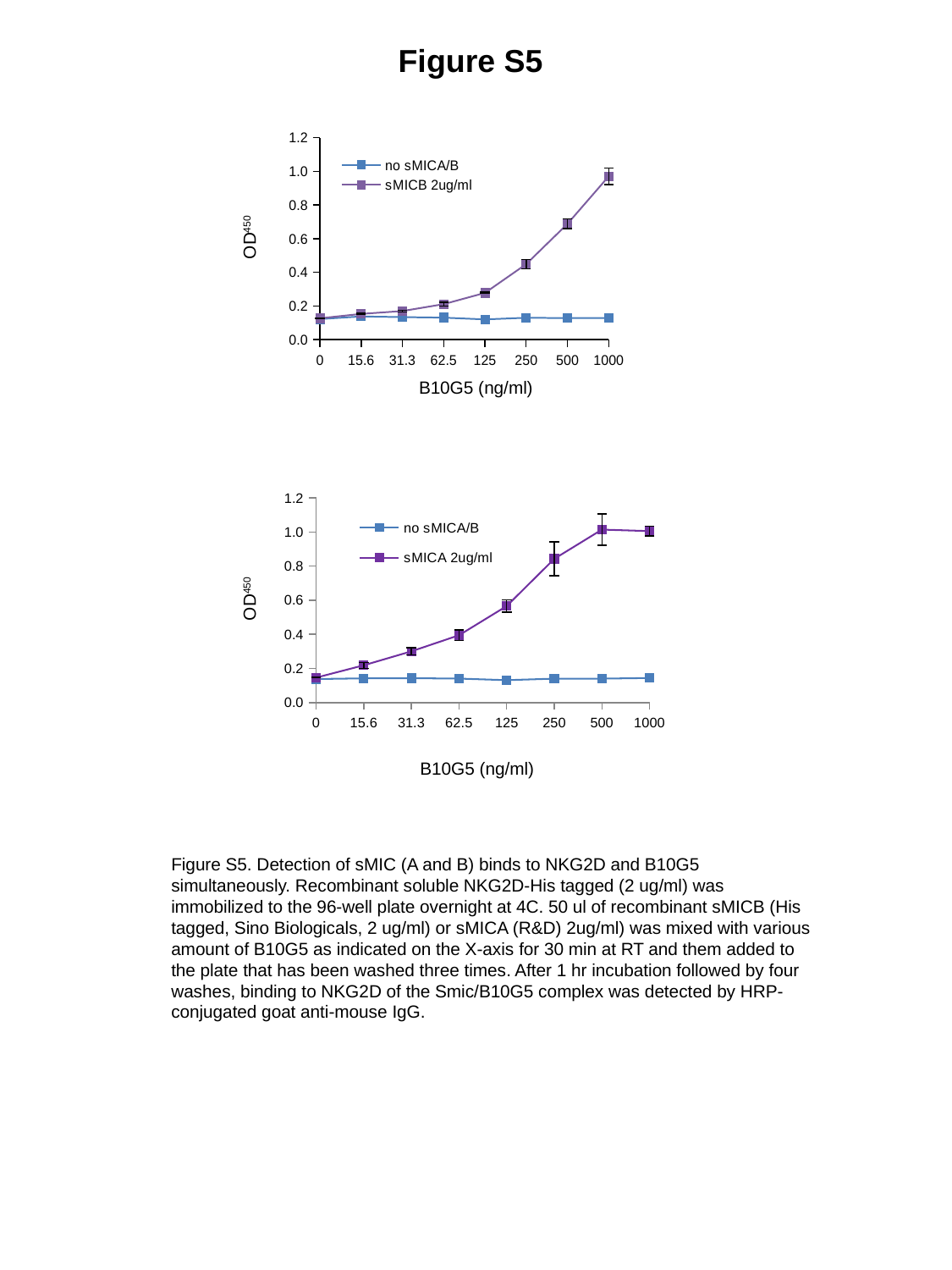

Figure S5
### Chart
| Category | no sMICA/B | sMICB 2ug/ml |
|---|---|---|
| 0 | 0.12231722863342342 | 0.1267904271276111 |
| 15.6 | 0.13772160343609782 | 0.15285351796497257 |
| 31.3 | 0.13395810222842022 | 0.16994534645339784 |
| 62.5 | 0.13066081539742025 | 0.21086350101300846 |
| 125 | 0.120233846066792 | 0.27785018943147083 |
| 250 | 0.12988418282375636 | 0.4485009869159054 |
| 500 | 0.12783940684674525 | 0.6883489836812456 |
| 1000 | 0.12838873178757815 | 0.9706692707504484 |OD450
B10G5 (ng/ml)
### Chart
| Category | no sMICA/B | sMICA 2ug/ml |
|---|---|---|
| 0 | 0.1377984412557137 | 0.14610453950247496 |
| 15.6 | 0.14189173990097748 | 0.218193202059507 |
| 31.3 | 0.14286016643306146 | 0.3003034483071138 |
| 62.5 | 0.14067762762935154 | 0.39465117404488415 |
| 125 | 0.13152886568268413 | 0.5655722600110195 |
| 250 | 0.14016072380193215 | 0.8430068340583423 |
| 500 | 0.14014284831352916 | 1.0144091727818223 |
| 1000 | 0.14393610958551675 | 1.0055347159828631 |OD450
B10G5 (ng/ml)
Figure S5. Detection of sMIC (A and B) binds to NKG2D and B10G5 simultaneously. Recombinant soluble NKG2D-His tagged (2 ug/ml) was immobilized to the 96-well plate overnight at 4C. 50 ul of recombinant sMICB (His tagged, Sino Biologicals, 2 ug/ml) or sMICA (R&D) 2ug/ml) was mixed with various amount of B10G5 as indicated on the X-axis for 30 min at RT and them added to the plate that has been washed three times. After 1 hr incubation followed by four washes, binding to NKG2D of the Smic/B10G5 complex was detected by HRP-conjugated goat anti-mouse IgG.

## Slide 6
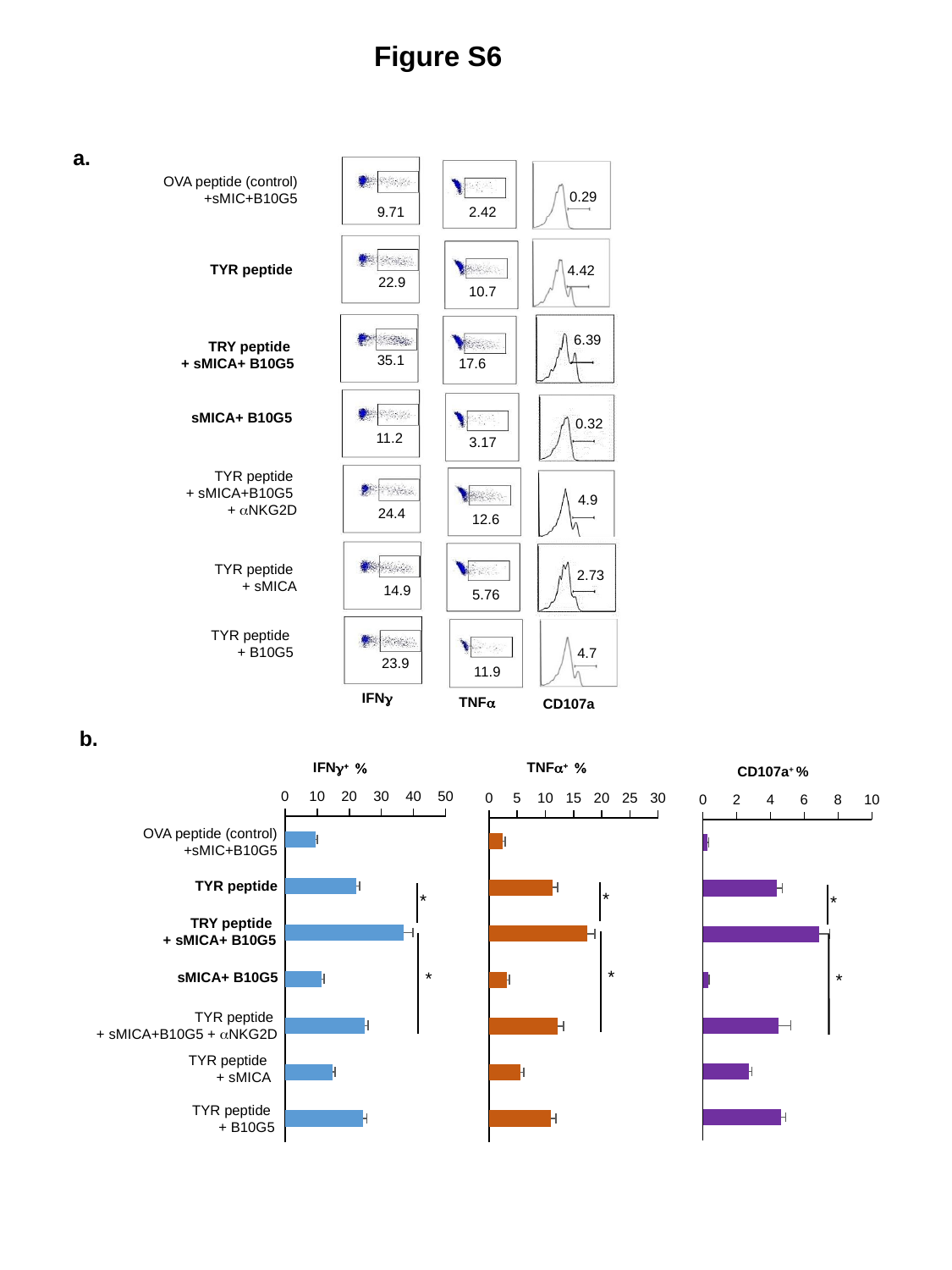

Figure S6
a.
2.42
10.7
17.6
3.17
12.6
5.76
11.9
TNFa
0.29
4.42
6.39
0.32
4.9
2.73
4.7
CD107a
 OVA peptide (control) +sMIC+B10G5
 TYR peptide
TRY peptide
+ sMICA+ B10G5
 sMICA+ B10G5
TYR peptide
+ sMICA+B10G5
+ aNKG2D
 TYR peptide
+ sMICA
TYR peptide
+ B10G5
9.71
22.9
35.1
11.2
24.4
14.9
23.9
IFNg
b.
TNFa+ %
IFNg+ %
CD107a+ %
### Chart
| Category | |
|---|---|
### Chart
| Category | |
|---|---|
### Chart
| Category | |
|---|---|OVA peptide (control) +sMIC+B10G5
TYR peptide
TRY peptide
+ sMICA+ B10G5
 sMICA+ B10G5
TYR peptide
+ sMICA+B10G5 + aNKG2D
 TYR peptide
+ sMICA
TYR peptide
+ B10G5
*
*
*
*
*
*

## Slide 7
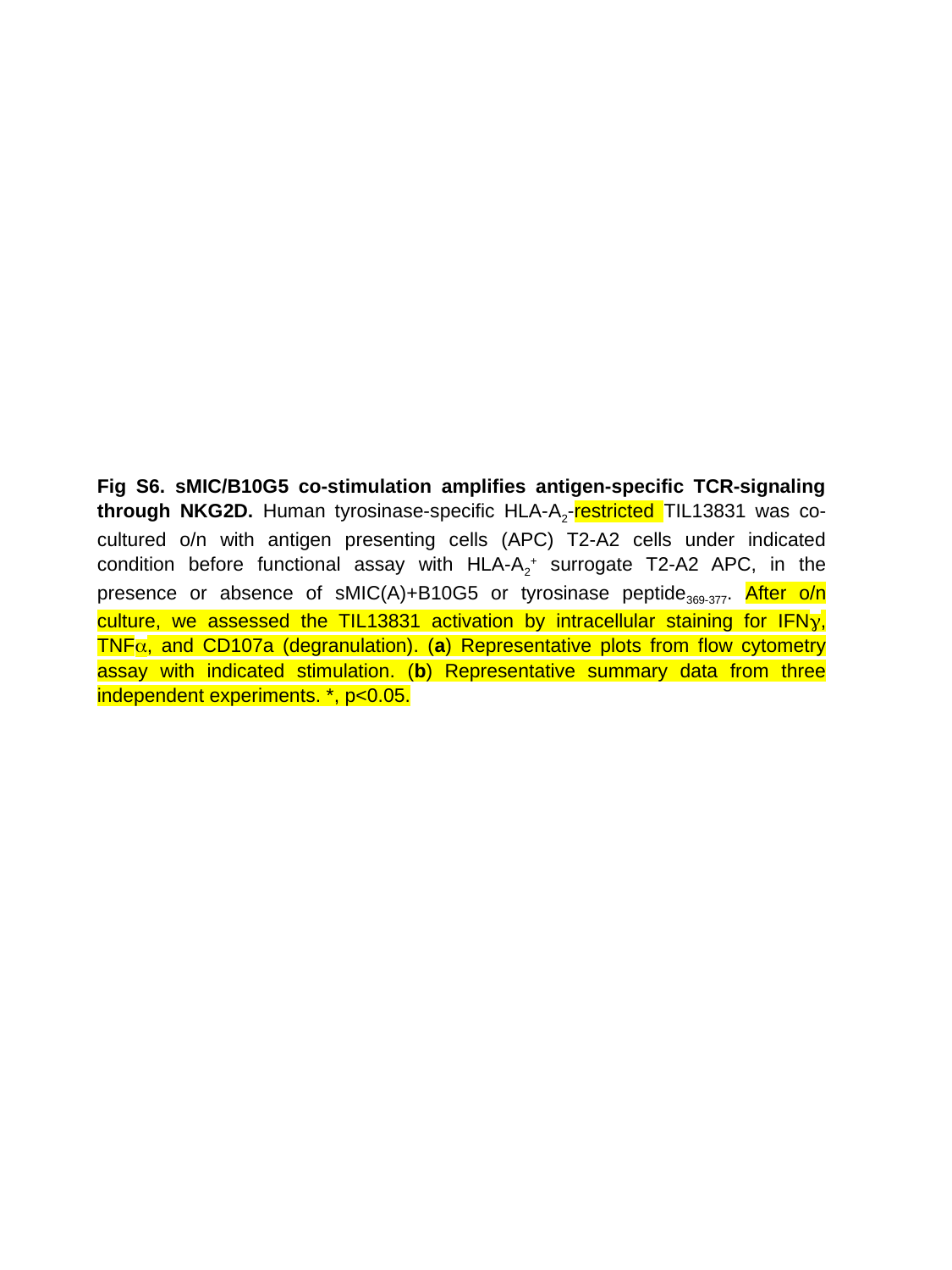

Fig S6. sMIC/B10G5 co-stimulation amplifies antigen-specific TCR-signaling through NKG2D. Human tyrosinase-specific HLA-A2-restricted TIL13831 was co-cultured o/n with antigen presenting cells (APC) T2-A2 cells under indicated condition before functional assay with HLA-A2+ surrogate T2-A2 APC, in the presence or absence of sMIC(A)+B10G5 or tyrosinase peptide369-377. After o/n culture, we assessed the TIL13831 activation by intracellular staining for IFNg, TNFa, and CD107a (degranulation). (a) Representative plots from flow cytometry assay with indicated stimulation. (b) Representative summary data from three independent experiments. *, p<0.05.

## Slide 8
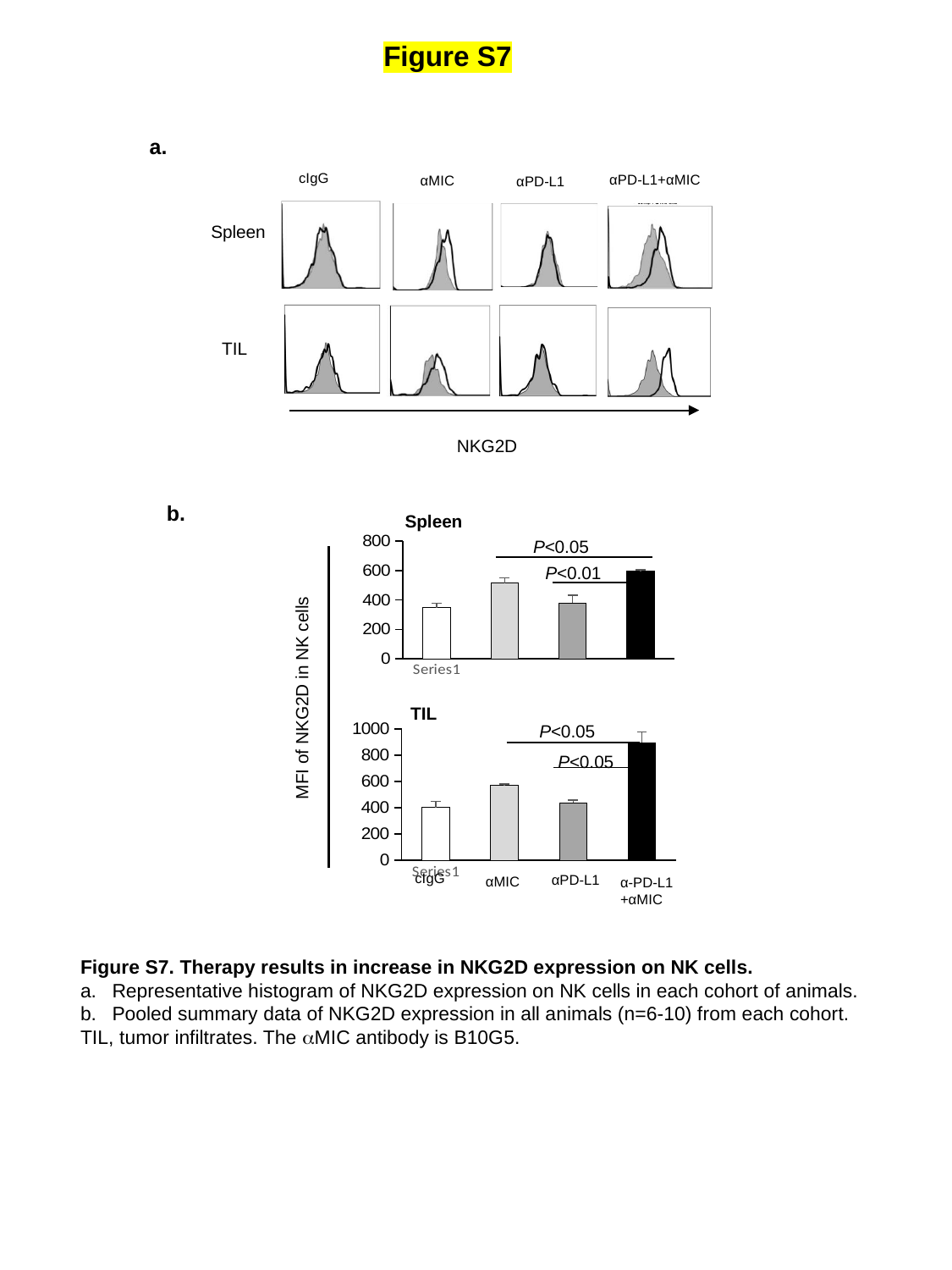

Figure S7
a.
cIgG
αPD-L1+αMIC
αMIC
αPD-L1
Spleen
TIL
NKG2D
b.
Spleen
P<0.05
### Chart
| Category | |
|---|---|
| | 347.0 |
| | 515.7143 |
| | 378.4 |
| | 594.0 |P<0.01
MFI of NKG2D in NK cells
TIL
P<0.05
### Chart
| Category | |
|---|---|
| | 401.3 |
| | 567.5 |
| | 435.0 |
| | 898.5 |P<0.05
cIgG
αPD-L1
αMIC
α-PD-L1
+αMIC
Figure S7. Therapy results in increase in NKG2D expression on NK cells.
Representative histogram of NKG2D expression on NK cells in each cohort of animals.
Pooled summary data of NKG2D expression in all animals (n=6-10) from each cohort.
TIL, tumor infiltrates. The aMIC antibody is B10G5.
